# Transcriptomics Analysis of *Crassostrea hongkongensis* for the Discovery of Reproduction-Related Genes

**DOI:** 10.1371/journal.pone.0134280

**Published:** 2015-08-10

**Authors:** Ying Tong, Yang Zhang, Jiaomei Huang, Shu Xiao, Yuehuan Zhang, Jun Li, Jinhui Chen, Ziniu Yu

**Affiliations:** 1 Key Laboratory of Marine Bio-resource Sustainable Utilization, Laboratory of Applied Marine Biology, South China Sea Institute of Oceanology, Chinese Academy of Sciences, Guangzhou, China; 2 South China Sea Bio-Resource Exploitation and Utilization Collaborative Innovation Center, Guangzhou, China; 3 Qingyuan Polytechnic, Qingyuan, China; The Ohio State University, UNITED STATES

## Abstract

**Background:**

The reproductive mechanisms of mollusk species have been interesting targets in biological research because of the diverse reproductive strategies observed in this phylum. These species have also been studied for the development of fishery technologies in molluscan aquaculture. Although the molecular mechanisms underlying the reproductive process have been well studied in animal models, the relevant information from mollusks remains limited, particularly in species of great commercial interest. *Crassostrea hongkongensis* is the dominant oyster species that is distributed along the coast of the South China Sea and little genomic information on this species is available. Currently, high-throughput sequencing techniques have been widely used for investigating the basis of physiological processes and facilitating the establishment of adequate genetic selection programs.

**Results:**

The *C*.*hongkongensis* transcriptome included a total of 1,595,855 reads, which were generated by 454 sequencing and were assembled into 41,472 contigs using *de novo* methods. Contigs were clustered into 33,920 isotigs and further grouped into 22,829 isogroups. Approximately 77.6% of the isogroups were successfully annotated by the Nr database. More than 1,910 genes were identified as being related to reproduction. Some key genes involved in germline development, sex determination and differentiation were identified for the first time in *C*.*hongkongensis* (*nanos*, *piwi*, *ATRX*, *FoxL2*, *β-catenin*, etc.). Gene expression analysis indicated that *vasa*, *nanos*, *piwi*, *ATRX*, *FoxL2*, *β-catenin* and *SRD5A1* were highly or specifically expressed in *C*.*hongkongensis* gonads. Additionally, 94,056 single nucleotide polymorphisms (SNPs) and 1,699 simple sequence repeats (SSRs) were compiled.

**Conclusions:**

Our study significantly increased *C*.*hongkongensis* genomic information based on transcriptomics analysis. The group of reproduction-related genes identified in the present study constitutes a new tool for research on bivalve reproduction processes. The large group of molecular markers discovered in this study will be useful for population screening and marker assisted selection programs in *C*.*hongkongensis* aquaculture.

## Introduction

Mollusks represent a major branch of lophotrochozoan organisms and have been an interesting target for the study of reproductive biology because diverse reproductive strategies have evolved in this phylum. Mollusks include dioecious and hermaphroditic species, and species that are capable of sex changes [1,2]. Studies on the reproduction of mollusk species may provide insights into the reproduction system and its evolution.

*Crassostrea hongkongensis*, a member of the phylum Mollusca, is a dominant oyster species on the coast of the South China Sea, with a cultivation history of ~700 years [3]. It is considered a popular seafood with a market demand that’s been growing over time, and it is one of the three major oyster species produced by aquaculture in China. In 2011, the production of *C*.*hongkongensis* reached 1,100,000 tons [4]. However, in recent years, the *C*.*hongkongensis* cultivation industry has been affected by the emerging shortage of seeds due to region planning, industrialization and urbanization in some seed-producing areas as well as population degradation. Therefore, there is urgent demand for better and higher seed production. The identification of reproduction-related genes will advance our understanding of *C*.*hongkongensis* reproduction and possibly contribute to the production of high-quality seeds. It will also help us understand the general underlying molecular mechanisms of bivalve reproduction, which will provide a basis for the development of aquaculture technology for bivalves.

To date, our knowledge of the molecular mechanisms of oyster reproduction is still limited. Several genes involved in germ cell development (*vasa* and *nanos*) have been identified in the Pacific oyster *C*. *gigas* and pearl oyster *P*. *fucata* [5,6]. Genes related to the sex determining pathways (*Dmrt-like*, *SoxH*, *FoxL2*and *fem-1like*) have been reported in *C*.*gigas* and *P*.*margaritifera* [7,8,9], and it has been shown that oysters share some key sex determination genes with vertebrates [7]. Hormones related to gonad development, oocyte maturation and vitellogenesis, including insulin [10], estradiol [11], 5-hydroxytryptamine (5-HT) [12] and gonadotropin-releasing hormone (GnRH) [13], and hormone receptors (estrogen receptor, 5-HT receptor, GnRH receptor) [14,15,16] have also been studied in oysters. Neuropeptides, which include egg-laying hormones (ELH) that control egg-laying behavior, were reported recently in *P*.*fucata* and *C*.*gigas* [17].

However, in-depth studies on the oyster reproduction mechanisms are hindered by the lack of genetic information. Although several studies have produced EST sequences in *C*.*hongkongensis* [18,19,20], gene sequences for this species are still scarce in GenBank. Recently, high-throughput next-generation sequencing (NGS) technologies, which include Solexa/Illumina, SOLiD/Applied technologies and 454/Roche, have created unprecedented opportunities for generating genetic information in non-model species and have been utilized to survey the genes related to reproduction. Roche’s 454 GS-FLX platform, which now provides a sequence read size approaching the length of conventional Sanger sequencing and sequencing coverage that is much deeper than that available with Sanger sequencing, has been widely used for de novo transcriptome sequencing in species including fish (turbot) [21]; shrimp (*Litopenaeus vannamei*) [22]; and mollusks (*Bathymodiolus azoricus* [23], *Ruditapes philippinarum* [24], *Patinopecten yessoensis* [25], and *Mytilus edulis* [26]). In particular, reproduction-related genes have been discovered in turbot and *P*. *yessoensis* by 454 pyrosequencing and have been useful for understanding the molecular process of reproduction in these species [21,25].

In the present study, we sequenced and assembled the *C*.*hongkongensis* transcriptome obtained by 454 sequencing, and studied the genes related to reproduction. The group of reproduction-related genes identified through this analysis constitutes a new tool for research on the bivalve reproduction process and provided insights into the origin and ancient character of the reproduction-related genes. We also characterized SNPs and SSRs to be employed for genetic improvement purposes. Hopefully, our study would be of great importance by providing resources for functional gene discovery, genetic and genomic studies on this species.

## Materials and Methods

### Sample Preparation

Adult *C*.*hongkongensis* were purchased from an aquaculture farm in Zhanjiang, Guangdong Province, China. Tissues including gill, adductor muscle, digestive gland, hemocytes, mantle, heart, and male and female gonads were dissected from sexually mature adult oysters. Embryos at the4-cell stage, blastula, trochophore and D-shaped larval stages were collected by filtration on a 30 μm mesh. All samples were homogenized in TRIzol (Invitrogen, Carlsbad, USA) and stored at -80°C (total RNA isolation), or fixed in 4% paraformaldehyde buffer (*in situ* hybridization). Isolated RNA was quality and quantity checked using electrophoresis and a Nanodrop 2000c Spectrophotometer (Thermo Scientific, USA).

### 454 cDNA Library Construction and Transcriptome Sequencing

To maximize the discovery of *C*.*hongkongensis* genes, a normalized cDNA library was constructed. RNA from each sample was combined into a single pool and mixed well (see detail in S1 Table).The SMART (Switching Mechanism At 5’ end of RNA Template) kit (BD Clonetech, Mountain View, CA) was used to retrotranscribe total poly-adenylated RNA. First-strand cDNA was synthesized with SMART Oligo Ⅱoligonucleotide (5’-AAGCAGTGGTATCAACGCAGAGTACGGGGG-3’) and a modified oligo-dT primer (5’-AAGCAGTGGTATCAACGCAGAGTACTTTTGTTTTTTTTTCTTTTTTTTTTVN- 3’). Double-strand cDNA was obtained from 1μl of the first-strand PCR reaction with SMART PCR primer (5’-AAGCAGTGGTATCAACGCAGAGT-3’). Then, the amplified dsDNA products were purified using the QIAquick PCR Purification Kit (Qiagen, USA) and normalized using the Trimmer-Direct Kit (Evrogen, Moscow). The quality of the normalized cDNA library was determined on a 1.4% agarose gel. Approximately 2.5 μg of the normalized cDNA pool was used for a titration run using one quarter of a plate on the Roche 454 GS FLX sequencer (Roche, Basel, Switzerland), and then 15 μg of cDNA was sequenced with a whole-plate run on the same equipment at the University of Illinois, USA. The reads from this sequencing were deposited in the NIH Short Read Archive database with Run accession number SRR949615.

### De Novo Assembly of the *C*.*hongkongensis* Transcriptome

GS FLX data were processed using the Roche GS FLX software (v2.6). *De novo* assembly of the transcriptome was performed using the GS De Novo Assembler v2.6 (Roche).The overlap settings used for this assembly were 40bp and 90% identity, with all other parameters set at the default values by the-cdna and-urt options. Prior to assembly, the primers and adaptors were trimmed accordingly.

### Sequence Annotation

Sequence annotation was performed against the Swiss-Prot and Non-redundant (Nr) protein databases, using BlastX (version2.2.28+) with a significance threshold cut off of E-value ≤ 1e-06. A maximum of 20 hits were taken into account for each blast query. To avoid redundant annotations, only the longest isotig from each isogroup was selected. Gene names were assigned to each selected isotig based on the best BLAST hit (highest score).

The set of longest isotigs was also used to identify Gene Ontology (GO) designations using the program Blast2GO [27].The BLAST result (xml file) from the Swiss-Prot annotation was imported into the Blast2GO program, and the GO terms associated with blast hits were retrieved. GO annotations were conducted only for isotigs with significant BLAST hits below an E-value of 1e-06, with 55 as the annotation cut-off and 5 as the GO weight. No HSP-hit coverage cut-off was used. InterproScan annotation was also conducted using Blast2GO.The obtained information on protein domains and motifs were included to improve global annotations.

Sequences of the longest isotig from each isogroup were uploaded to the online KEGG Automatic Annotation Server (KAAS; http://www.genome.jp/tools/kaas/) for ortholog assignment and pathway mapping. The bi-directional best hit (BBH) method was used to compare the *C*.*hongkongensis* transcriptome against each genome in the reference set of the KEGG GENES database.

### RT-qPCR

Total RNA was isolated from mantle, gill, heart, hemocytes, adductor muscle, digestive gland, male and female gonads (each sample was pooled from five individuals). After the digestion of genomic DNA with DNase I to prevent genomic DNA contamination, 1μg of total RNA was reverse transcribed using PrimeScript RT reagent Kit with gDNA eraser (Perfect Real Time) (Takara, Japan).The resulting cDNAs were diluted, and amount equivalent to 5 ng of starting RNA was assayed for reproduction-related gene expression using EF1α as the reference gene. SYBR-based qRT-PCR reactions (Light Cycler 480 Master Mix I, Roche, Basel, Switzerland) were performed on a Light Cycler 480 system (Roche, Basel, Switzerland) using the following reaction conditions: 95°C for 1 m in followed by 45 cycles of 95°C for 10 s, 58°C for 10 s and 72°C for 10 s. The primers used are listed in S2 Table. A melting curve was generated at the end of the reaction to check for the accurate amplification of the target amplicon. The PCR efficiencies of the target gene and reference gene were verified and were approximately equal. The relative gene expression was calculated according to the formula: 2 ^-(Ct target gene–Ct reference gene)^. Water was used instead of cDNA as a negative control for amplification, and DNAase-untreated cDNA was used to check for the absence of genomic DNA contamination. All the results are expressed as the mean ± s.e.m (standard error to the mean). The results were analyzed for statistical significance using one-way ANOVA (significance was considered when p < 0.01). Data were analyzed using the GraphPad Prism software version 5.0. The relative expression data of sex determination/differentiation genes were imported into the MultiExperiment Viewer software (http://www.tm4.org/mev.html) for heatmap production.

### mRNA *in situ* hybridization

A713-bp fragment for *Ch-nanos* and a1146-bp fragment for *Chpiwil1*were amplified by RT-PCR with the primers listed in S2 Table. These fragments were subcloned into the pGEM-easy T vector (Promega, USA) and sequenced. The resulting plasmids were linearized using SacII and NdeI in separate reactions. The linearized products were purified and used as a template to generate sense and antisense DIG-labeled RNA probes using a DIG RNA labeling kit (SP6/T7; Roche Diagnostics, Germany).

Ovaries of early developing stage, late developing stage and mature stage were fixed in 4% paraformaldehyde buffered with 1×phosphate saline (pH7.4) at 4°C overnight. Paraffin-embedded ovary samples were sectioned (7 μm thick) and mounted on glass slides coated with 1% Poly-L-Lysine solution. Slices were deparaffinized through two washes of xylene, hydrated through an ethanol gradient, washed with 1×PBS and then digested with proteinase K (10 μg/ml) for 10 min at 37°C. Following wash and prehybridization, the serial sections were hybridized overnight at 60°C with either antisense or sense probes in hybridization solution. After an extensive wash, the sections were incubated in 1% blocking reagent in PBS for 1 hr and then transferred into 1/5000 dilution of anti-DIG alkaline phosphatase conjugated fab fragment at room temperature for 2 hrs. A mixture of BCIP/NBT was then used for color development. All slices were mounted with Mowiol and imaged under a Leica DMR HC (Leica, Germany) microscope.

### Sequence and Phylogenetic Analysis

Sequences from various organisms (see S3 Table for accession numbers) were obtained from the NCBI genome server (http://blast.ncbi.nlm.nih.gov/Blast.cgi) and aligned using the DNAMAN 8 program (http://www.lynnon.com/). The optimal phylogenetic tree of each gene was constructed using the neighbor-joining method, implemented in the MEGA5.0 program [28]. One thousand boot strap trials were run for each node. The protein motifs were analyzed with SMART (http://smart.embl-heidelberg.de/smart/set_mode.cgi?NORMAL=1).

### SNP and SSR Discovery

Potential SNPs were detected using GS Reference Mapper v2.6 with default parameters (cDNA mode). The set of the longest isotig was used as a reference sequence. Primers and adaptors were trimmed before mapping. SNP identification was limited to isotigs or contigs: (i) the total number of reads that fully span the difference location (Total Depth) ≥8; (ii) the percentage of different reads versus total reads that fully span the difference location (Var Freq) ≥ 25%.For SSR detection, the set of longest isotig was fed into the MicroSAtellite (MISA) program [29]. All types of SSRs, from dinucleotides to hexanucleotides, were searched using default settings (the minimum repeat number was six for dinucleotide and five for tri-, tetra-, penta- and hexanucleotide).

## Results and Discussion

### 454 Transcriptome Sequencing and Assembly

The normalized pooling library was prepared from larvae of four different developmental stages (4-cell stage, blastula, trochophore and D-shaped larvae) and various tissues (gill, adductor muscle, digestive gland, hemocytes, mantle, heart, ovary and testis) and sequenced using the Roche 454 GS FLX sequencer. A single run of the 454 sequencing generated 1,595,855 raw sequencing reads with an average length of 382 bp and total output of 609,282,210 bp (Table 1).

**Table 1:** Summary statistics of the transcriptome assembly for *Crassostrea hongkongensis*.

Category	Value
Total number of raw reads	1,595,855
Total length of raw sequences	609,282,210 bp
Total number of clean reads	1,405,240
Total length of clean reads(bp)	522,591,765
reads assembled	1,266,466
**Total contigs**	**41,472**
Average length of contigs	958bp
Contig size N50	1,571bp
**Total isotigs**	**33,920**
Average length of isotigs	1,924bp
Isotig size N50	2,752bp
**Total Isogroups**	**22,829**
Total singletons	138,631
Total unigenes	161,460

The 41,472 contigs were assembled by GS De Novo Assembler v2.6, with an average length of 958 bp (Table 1).These contigs were then joined into 33,920 isotigs with an average length of 1,492 bp (N50 = 2,752 bp), and the length of 21,830 isotigs (64.4% of total isotigs) were longer than 1,000 bp (S1 Fig). The isotigs were further grouped into 22,829 isogroups. The remaining 138,631 reads that were not assembled by the GS De Novo Assembler v2.6 were considered singletons. In total, 161,460 unigenes (#isogroups + #singletons) were produced. Because previous reports indicated that most singletons represent lowly expressed transcripts, artifacts derived from cDNA synthesis, sequencing and contamination [30], they were excluded from the following analysis in this study.

Our 454 sequencing data and GS *De Novo* assembly of *C*.*hongkongensis* transcriptome were compared favorably with transcriptomes from other mollusks (produced with NGS). The average length of the raw reads and contigs in *C*.*hongkongensis* transcriptome was 382 bp and 958 bp, respectively, which are better than or similar to those of molluscan transcriptomes obtained from 454 sequencing: 283 bp and 509 bp in *B*.*azoricus* [31], 369 bp and 535 bp in *L*.*elliptica* [32], 319 bp and 723 bp in *C*.*angulata* [33], 313 bp and 618 bp in *P*.*yessoensis* [25]. They were better than those obtained from Illumina: 61 bp and 536 bp in *Radix balthica* [34], 90 bp and 874 bp in *C*.*virginica* [35]. Overall, the *C*.*hongkongensis* transcriptome obtained from 454 sequencing here is a high-quality one in either sequencing or assembly.

### Gene Annotation and Functional Classification

To identify the putative functions of the *C*.*hongkongensis* genes, the longest isotig from each isogroup was chosen as a representative and compared with those in Swiss-prot protein database and NCBI Non-redundant (Nr) database. A total of 12,273 isotigs (53.8% of total isogroups) showed significant matches to known proteins in the Swiss-prot database, corresponding to 10,079 different well-annotated proteins (S4 Table). A higher percentage of isogroups (17,721 isotigs, 77.6% of total isogroups) had significant matches to known proteins in the Nr database (S4 Table), corresponding to 13,631 unique proteins. The remaining isogroups that were not annotated appeared to be either *C*.*hongkongensis*-specific genes or homologous genes with unknown functions in other species.

Gene Ontology (GO), which is an international standardized gene functional classification system, was used to classify the predicted *C*.*hongkongensis* genes in terms of their associated biological processes, cellular components and molecular functions. GO terms were retrieved from the association to best-hit for 9,909 (43.4%) of the overall 22,829 isogroups. Protein domain and motif information were retrieved by InterProScan via Blast2GO, and corresponding annotations were merged with already existing GO terms. A total of 15,050 isogroups provided significant InterProScan information, with 7,498 of them resulting in GO annotation. After merging, a total of 11,633 isogroups (51.0% of total isogroups) were assigned at least one GO term. In the biological process category, genes involved in the cellular process (GO:0009987) and metabolic process (GO:0008152) were prominently represented. Cells (GO:0005623) and organelles (GO:0043226) represented the majority of terms in the cellular component category, whereas in the molecular function category, the vast majority is related to binding (GO:0005488) and catalytic activity (GO:0003824; Fig 1). The GO classification results in each category here were similar to the previously sequenced *P*.*yessoensis* and *Chlamys farreri* via 454 sequencing [25,36].

**Fig 1:**
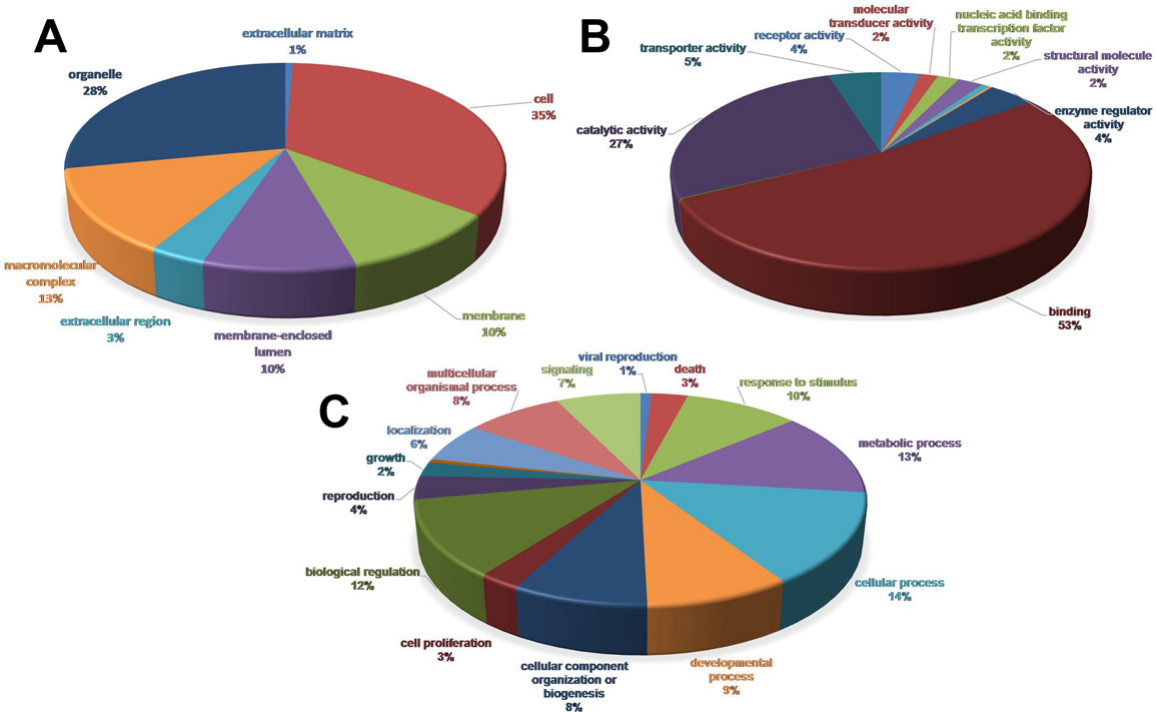
Gene Ontology(GO) analysis of the *Crassostrea hongkongensis* transcriptome on level 2. The percentage and distribution of top-level GO-terms were portrayed in the three categories: (A) Cellular component; (B) Molecular function and (C) Biological process.

**Fig 2:**
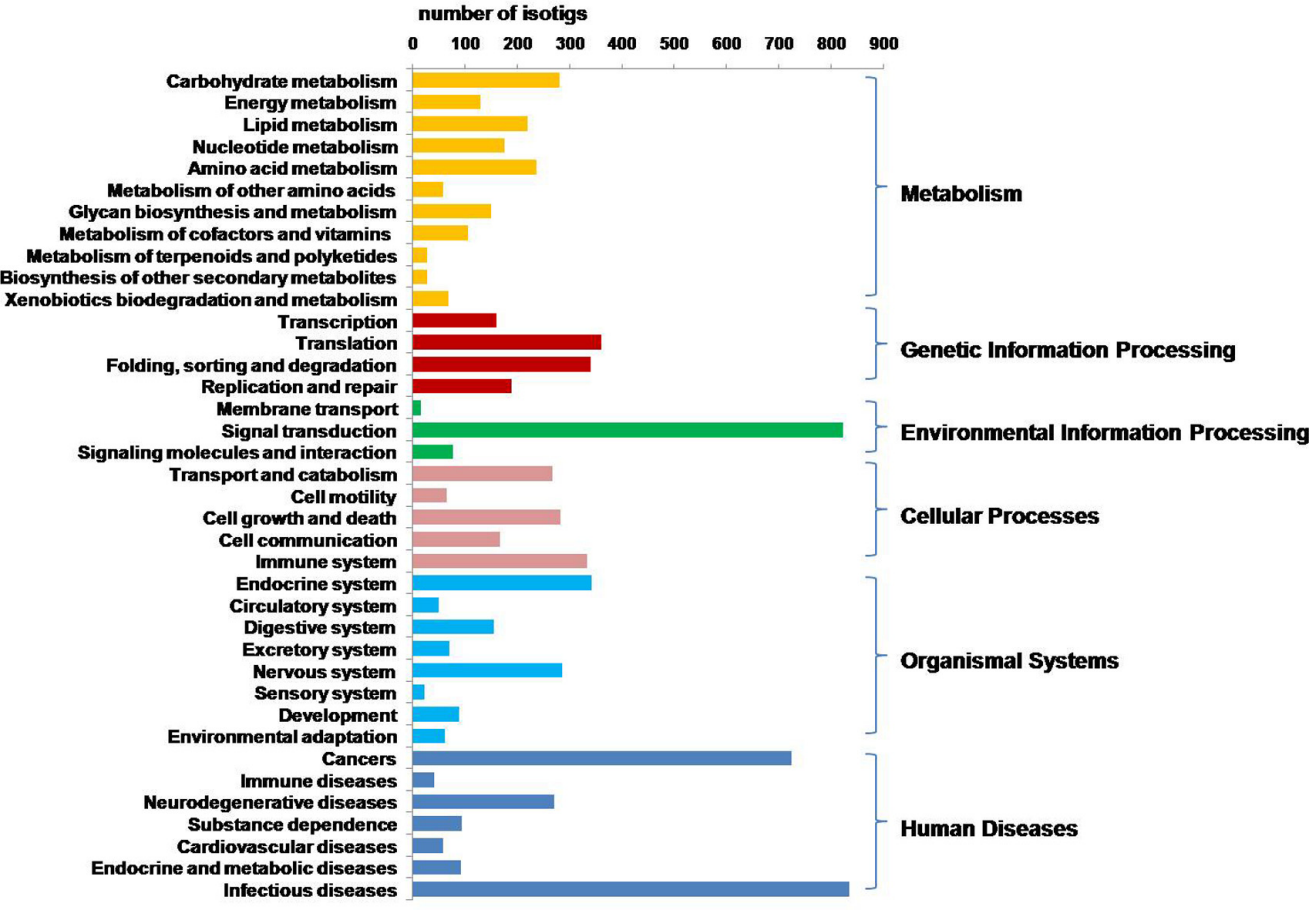
KEGG biochemical mappings for *C*. *hongkongensis* transcriptome.

The KEGG orthology (KO) is a classification system that provides an alternative functional annotation of genes based on their associated biological pathways. The KO annotations for *C*.*hongkongensis* were based on sequence similarity searches for reference sequences in the NCBI database. Overall, 5,197 isogroups were assigned to KOs, which are involved in 257 different pathways (S5 Table). The most populated pathways were “infectious diseases” with 835 isogroups involved, “signal transduction” with 823 isogroups and “cancers” with 725isogroups (Fig 2). The KEGG pathways distribution in the *C*.*hongkongensis* transcriptome was identical to those in the *Mizuhopecten yessoensis* and *C*. *virginica* transcriptomes [35,37].

Overall, the annotation and functional classification of *C*.*hongkongensis* transcripts provided a valuable dataset for the identification of functional genes, investigating specific bioprocesses and pathways as well as for further genome-wide research and analyses in this species.

### Genes and Pathways Related to Reproduction

Reproduction is a biological process by which new individual orgamisms–“offspring”–are produced from “parents”. The reproduction process can be divided into the following categories: germline development, sex determination/differentiation, oocyte maturation and spawning, fertilization and others. Both GO and KEGG analyses identified transcripts that are potentially involved in reproduction. GO classification identified 1910 genes related to reproduction (GO:0000003, S6 Table), which was much more than those (318 reproduction-related genes) identified in *P*. *yessoensis* by 454 sequencing [25]. KEGG annotation identified 205 genes related to reproduction, distributed in 8 pathways (Table 2). These genes covered all major processes of reproduction, including germline development, sex determination/differentiation, oocyte maturation, fertilization and others. Because germline development, sex determination/differentiation and oocyte maturation are basic processes of reproduction for *C*.*hongkongensis*, genes functioning in these processes were analyzed in priority below.

**Table 2:** Reproduction-related pathways identified in the *C*. *hongkongensis* transcriptome.

Description	KEGG code	No. of genes in the pathway
Insulin signaling pathway	ko04910	56
Oocyte meiosis	ko04114	50
Progesterone-mediated oocyte maturation	ko04914	41
Estrogen signaling pathway	ko04915	35
GnRH signaling pathway	ko04912	32
Prolactin signaling pathway	ko04917	26
Ovarian Steroidogenesis	ko04913	15
Steroid hormone biosynthesis	ko00140	12

### Germline development genes

The 84 genes related to germline development were identified by GO analysis (GO:0007281, S6 Table) and literature supported searching (S7 Table). Table 3 shows some relevant genes with well-known functions in the process, and most of them were identified for the first time in *C*.*hongkongensis*, including some core germline genes such as *vasa*, *nanos* and *piwi*.

**Table 3:** Selection of some novel germline development genes identified in the *C*.*hongkongensis* transcriptome.

Gene name	Isotig name	Isotig length	E-value	Species	Gene ontology
*Bmp4*	isotig19635	1998	3.36E-78	*Gallus gallus*	GO:0008083 GO:0007281
*Bru*	isotig17186	3483	1.85E-107	*Danio rerio*	GO:0007283 GO:0007281
	isot ig22487	1373	2.37E-98	*Danio rerio*	GO:0007286 GO:0008016
*gcl*	isotig19380	2076	2.06E-145	*Mus musculus*	GO:0007275 GO:0007277
*lin28*	isotig22508	1370	8.70E-44	*Drosophila melanogaster*	GO:0007281 GO:0007549
*mago*	isotig30591	618	6.46E-90	*Homo sapiens*	GO:0008103 GO:0007267
*mex-3*	isotig16497	5168	3.69E-79	*Xenopus laevis*	GO:0008270 GO:0005509
*nanos*	isotig23837	3166	2.13E-175	*Mus musculus*	GO:0007444GO:0007314
*par-1*	isotig31179	583	2.47E-09	*Caenorhabditis elegans*	
*piwi*	isotig17193	3468	0	*Mus musculus*	GO:0003729 GO:0005654
*pum*	isotig11287	3581	1.84E-114	*Rattus norvegicus*	GO:0007291 GO:0007475
	isotig16484	5232	0	*Mus musculus*	GO:0003730 GO:0005829
*stau*	isotig16834	4044	6.56E-108	*Rattus norvegicus*	GO:0003723 GO:0005875
*tud*	isotig09805	3781	5.31E-18	*Homo sapiens*	GO:0005737GO:0000003
	isotig16579	4788	1.23E-12	*Homo sapiens*	GO:0009987GO:0000003
	isotig16345	6854	4.22E-47	*Oryzias latipes*	GO:0005515GO:0007275
	isotig17301	3335	2.93E-30	*Homo sapiens*	GO:0005739GO:0005515
	isotig18032	2692	4.05E-28	*Oryzias latipes*	GO:0009987GO:0005737
	isotig22822	1318	3.43E-11	*Homo sapiens*	
*vasa*	isotig11445	3166	2.13E-175	*Mus musculus*	GO:0007281GO:0007283

**Fig 3:**
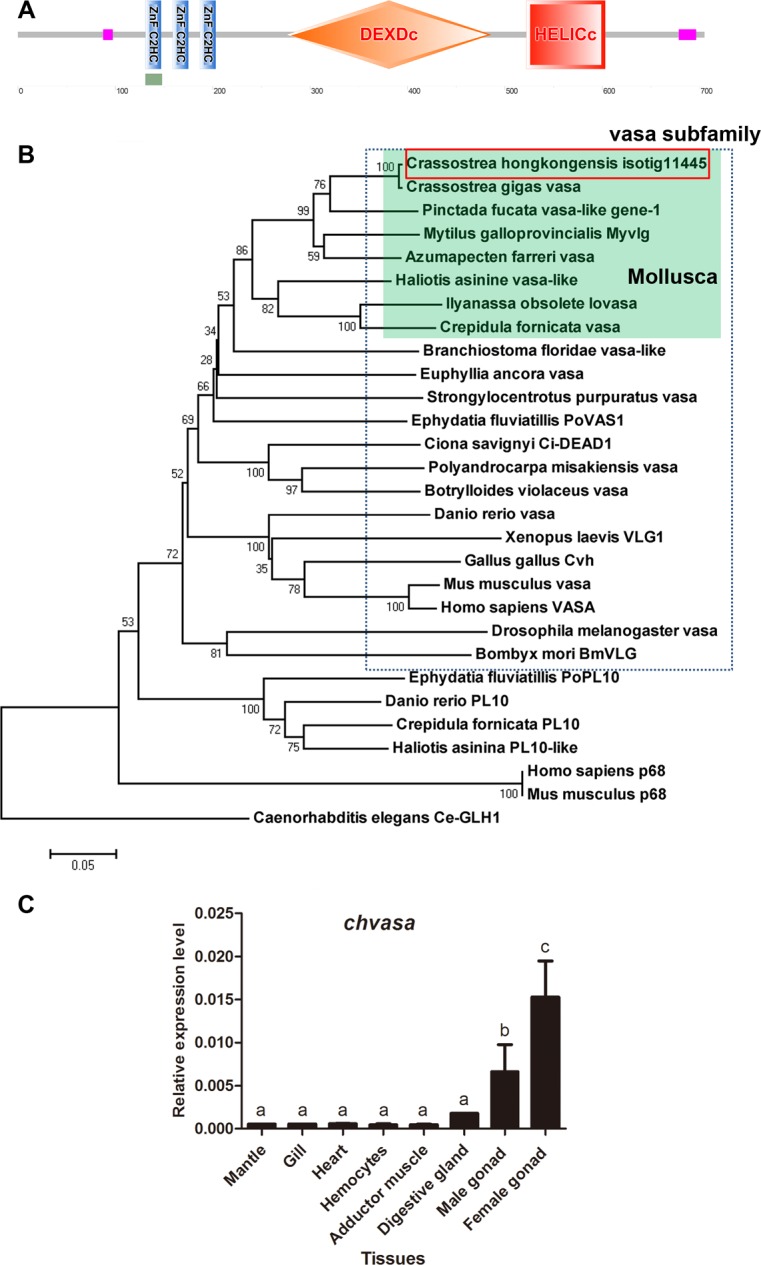
Vasa gene identified in the *Crassostrea hongkongensis* transcriptome and its expression profile. (A) Domain structure of the *C*.*hongkongensis vasa* ortholog. (B) Molecular phylogenetic tree of Vasa subfamily and related proteins. Bootstrap values from 1000 trials are indicated at each branch node. The scale bar indicates 0.05 amino acid replacements per site. *C elegans* Ce-GLH-1 was used as an outgroup. The transcript encoding *Chvasa*, indicated by a red open box, is properly aligned to the clade of molluscan Vasa colored by green. The Vasa subfamily proteins are enclosed by the dotted lines. For the GenBank accession numbers of the reference sequences, see S3 Table. (C) Expression profile of *C*.*hongkongensis vasa* ortholog (*Chvasa*) in adult tissues with mean ± s.e.m. as error bars (n = 5).

*Vasa* is a key determinant in germline formation in eukaryotes. It encodes an RNA helicase that is characterized by the presence of zinc knuckle motifs, a DEAD box helicase domain and a helicase conserved C-terminal domain. In the *C*.*hongkongensis* transcriptome, a transcript of 3,166 bp (isotig11445) encoding a Vasa-related protein was identified and showed an ORF of 2,118 bp. The deduced amino acid sequence is 705 aa long and contains three zinc knuckle motifs, a DAED box helicase domain and a helicase C domain (Fig 3A). Phylogenetic analysis performed with related proteins indicated that the Vasa-related protein encoded by isotig11445 clustered to molluscan Vasa, particularly to bivalve Vasa (Fig 3B). Therefore, we named the *C*.*hongkongensis* Vasa-related protein “Chvasa”. *Chvasa* was highly expressed in female and male gonads with weak expression in other tissues (Fig 3C). In bivalves, a *vasa* ortholog has been characterized in *C*. *gigas* (oyster vasa-like gene, *Oyvlg*), and its expression was restricted to germline cells both in males and females, including germinal stem cells and auxiliary cells [38]. Knockdown of *Oyvlg* by RNAi resulted in germ cell underproliferation and prematurely arrested meiosis, indicating a key role of *vasa* in bivalve germ cell development [38,39]. Our experiment showed that a high expression level of *Chvasa* was detected in both female and male gonads, indicating a role for *Chvasa* in reproduction and possibly in the development and maintenance of germ cells. Further investigations are needed to understand the role of *Chvasa* in *C*.*hongkongensis* germline development.

**Fig 4:**
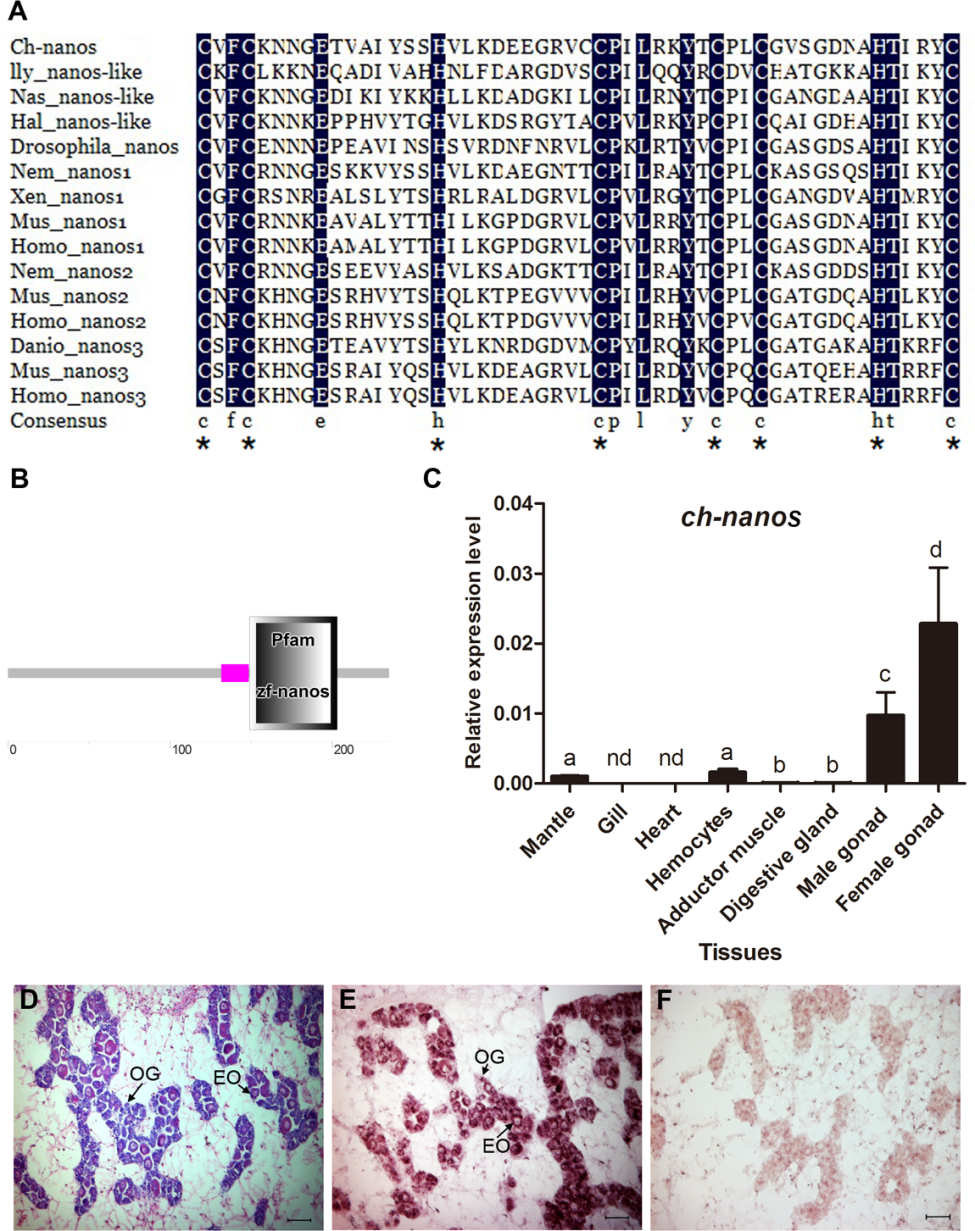
Analysis of Nanos ortholog identified in *C*.*hongkongensis* transcriptome. (A) Amino acid sequence alignment of the nanos RNA-binding domain predicted in the *Ch-nanos* with that of other Nanos orthologs. Two highly conserved CCHC zinc finger motifs are indicated by asterisks. For the GenBank accession numbers of the reference sequences, see S3 Table. (B) Domain structure of *Ch-nanos*. (C) Expression profile of in *Ch-nanos* adult tissues by qRT-PCR with mean±s.e.m. as error bars (n = 5). nd: not detected. (D) Histological analysis of *C*.*hongkongensis* female gonad at early developing stage. (E) Expression profile of *Ch-nanos* in early developing stage female gonad detected by ISH with antisense and sense probe. (F) Positive cells are stained in purple.OG: oogonia; EO: early vitellogenic oocyte.

*Nanos*, another conserved core germline gene, is a translational repressor characterized by a *nanos* RNA-binding domain with two conserved Cys-Cys-His-Cys zinc finger motifs. It plays important roles in early development and, more specifically, in primordial germ cell (PGC) development [40]. A transcript encoding a *nanos* ortholog (isotig23837) was identified in the *C*.*hongkongensis* transcriptome, with an ORF of 708 bp. The deduced amino acid sequence is 235 aa and contains a *nanos* RNA binding domain. Alignment of the amino acid sequence of the predicted *C*.*hongkongensis nanos* RNA binding domain with other orthologs indicated high conservation of two characteristic CCHC zinc finger motifs (Fig 4A and 4B). This *C*.*hongkongensis nanos* mRNA was named “*Ch-nanos*”.*Ch-nanos* was specifically expressed in both male and female gonads when examined by RT-qPCR, suggesting an important role of *Ch-nanos* in *C*.*hongkongensis* reproduction. The expression level of *Ch-nanos* in female gonads was 2.3-fold higher than that in male gonads (Fig 4C). mRNA *in situ* hybridization showed that *Ch-nanos* was specifically expressed in developing germ cells including oogonia and early vitellogenic oocytes, but not in somatic cells (Fig 4D–4F), indicating a possible role of *Ch-nanos* in the formation of germ cells; it might be used as a molecular marker for germ cells in early developing stage female gonads. In mollusks, *nanos* orthologs have been isolated in *Ilyanassa obsolete* [41], *Haliotis asinine* [42] and *P*.*fucata* [6]. In these studies, the expression patterns of molluscan *nanos* orthologs during embryonic and larval development have been reported. However, the expression patterns and functions of molluscan *nanos* orthologs in germline development are unclear. Here, we identified a *nanos* homologue in *C*.*hongkongensis* and studied its expression pattern in different tissues and during germ cell development. It will be interesting to further follow the expression pattern of *Ch-nanos* during germline development and study its specific role in germ cells.

**Fig 5:**
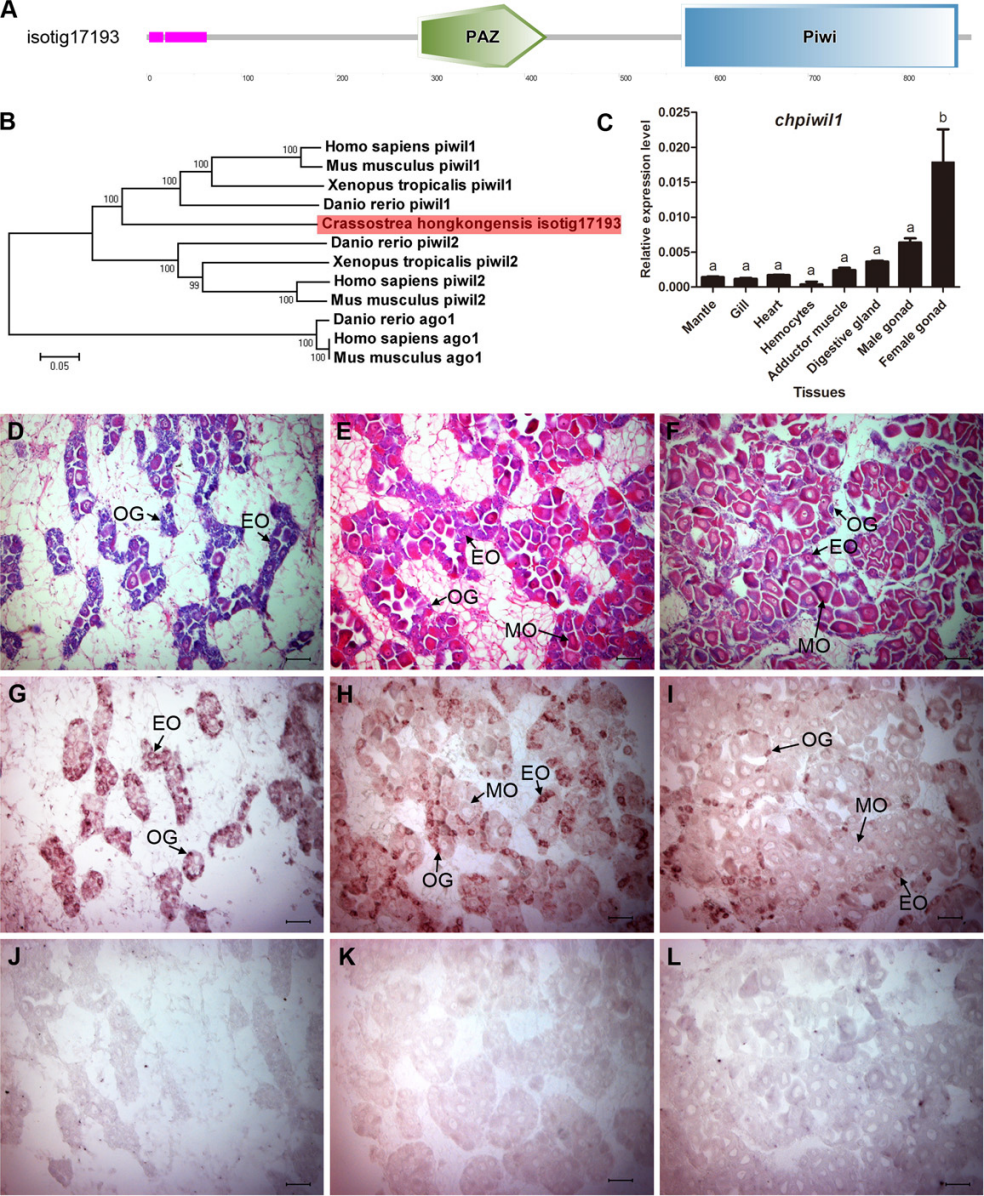
Analysis of Piwi genes identified in *C*.*hongkongensis* transcriptome. (A) Domain structure of the *C*.*hongkongensis Piwi* orthologs. (B) Phylogenetic tree constructed using the neighbor-joining method on the basis of the amino acid sequences alignment of *C*.*hongkongensis Piwi* ortholog with Piwi-like proteins of vertebrates. A total of 1000 bootstrap trials were run. Bootstrap values were indicated at each branch node. The scale bar represents an evolutionary distance of 0.1 amino acid substitutions per position. The transcript encoding *C*.*hongkongensis Piwi* ortholog, colored by red, was aligned to the clade of vertebrate Piwil1proteins. For the GenBank accession numbers of the reference sequences, see S3 Table. (C) Expression profile of *ChPiwil1* in adult tissues by qRT-PCR with mean±s.e.m. as error bars (n = 5). (D-L) Expression profile of *ChPiwil1*during *C*.*hongkongensis* oogenesis by ISH. Histological analysis of *C*.*hongkongensis* female gonad at early developing stage (D), late developing stage (E) and mature stage (F) stained with HE. Expression profile of *ChPiwil1*during *C*.*hongkongensis* oogenesis by ISH with antisense (G-I) and sense probe (J-L). Positive cells are stained with brown or purple. OG: oogonia; EO: early vitellogenic oocyte; MO: mature ova.

*Piwi* (P-element induced wimpy testis) is a member of the argonaute family of small RNA-binding proteins that possesses a PAZ domain in the middle and a PIWI-domain at the C-terminal end. In *Drosophila* and *C*.*elegans*, the lack of Piwi results in complete depletion of germline stem cells and sterility in both males and females. In contrast, overexpression of *piwi* increases both the number and mitosis rate of germline stem cellsin *Drosophila* [43,44]. A PAZ domain and PIWI-domain containing transcript (isotig17193) was identified in the *C*.*hongkongensis* transcriptome (Fig 5A). Phylogenetic analysis revealed that isotig17193 was a close relative of vertebrate Piwi-like 1 protein (Piwil1) rather than other members of the Piwi-like protein family and Ago1 (Fig 5B). Isotig17193 was named “*ChPiwil1*”. *ChPiwil1*was expressed in gonads but at a low level in somatic organs (Fig 5C). Its expression in the ovary was 2.8-fold higher than that in the testis. We then studied the expression patterns of *ChPiwil1* during oogenesis using mRNA *in situ* analysis. We found that *ChPiwil1*was only expressed in developing germ cells such as oogonia and early vitellogenic oocytes, but not in mature oocytes (Fig 5D–5L). These results support a possible role of *ChPiwil1* in female germline stem cell development. In mollusks, information on *piwi* homologues is still scarce. To our knowledge, this is the first time that a *piwi* homologue and its expression pattern during germline development were reported in mollusk. Further investigations are needed to demonstrate the precise role of *piwi* genes in molluscan germline development.

Overall, the analysis of the *C*.*hongkongensis* transcriptome identified 84 germline development related genes. Among these, *vasa*, *nanos* and *piwi* are the three conserved core germline genes. The presence of *vasa*, *nanos* and *piwi* in *C*.*hongkongensis* indicates that it may share a conserved set of core genes with other animals for the generation of new germ cells in development.

### Sex-determination/differentiation genes

Sex determination and sex differentiation are two major processes that occur during sexual development. While the sex determination process determines whether the bipotential primordium will develop into a testis or an ovary, the sex differentiation process occurs immediately after the sex determination process and involves the actual development of the testes or ovaries from the undifferentiated gonad [45].GO analyses of ‘Sex determination’ and ‘sex differentiation’ term annotation identified only 4 isotigs as orthologues of the GO0007530 (sex determination, S6 Table) annotation and 11 isotigs as orthologues of the GO0007544 (sex differentiation, S6 Table) annotation.

We then searched the *C*.*hongkongensis* transcriptome for such genes that were previously identified in model organisms (Table 4, S8 Table). Of the 49 genes examined, homologues were found for 20 genes, indicating that *C*.*hongkongensis* share some common sex determination/differentiation genes with other animals on the sequence level. To our surprise, we did not find homologues of vertebrate *Dmrt1* (*Doublesex and MAB-3 related transcription factor 1*) and *Sry* (*sex-determining region on the Y-chromosome*), although we found homologues for other Dmrt and Sox family members (e.g., *Dmrt2a*, *Sox4*, *Sox6* and *Sox8*). *Dmrt1* is a transcription factor that contains a zinc finger DNA-binding motif (DM domain) and plays conserved roles in male sex determination and differentiation [46]. Members of this family also include the *doublesex* (*dsx*) gene in *Drosophila* and *MAB-3* in *C*.*elegans*. Recently, an oyster *Dmrt1* homologue was identified in *C*.*gigas* (*CgDsx*) and was found to be exclusively expressed in gonads [7]. *Sry* is a member of the Sox (Sry-related HMG box) protein family and is the single genetic trigger that regulates male sex determination and testicular differentiation [47]. A gene with a close relative to *Sox30* and *Sry* in vertebrates has been reported in the *C*.*gigas* genome (*CgSoxH*) and was exclusively expressed in testis [7]. The absence of *Dmrt1* and *Sry* homologues in our analysis may be a result of the low expression level of these genes in our samples or an artifact due to the incompleteness of the *C*.*hongkongensis* transcriptome dataset.

**Table 4:** Presence of sex determination/differentiation genes from *Caenorhabditis elegans*, *Drosophila melanogaster*, *Danio rerio*, *Mus musculus* and *Crassostrea hongkongensis*.

Gene common name	Species with homologues (homologue names)				
	Fly (D)	Worm (C)	Fish (Dr)	Mouse (M)	Oyster(Ch)
*WT1*			yes	yes	
*Sf1*			yes	yes	
*CBX2*	yes		yes	yes	
*LHX9*			yes	yes	yes
*EMX2*			yes	yes	
*GATA4*			yes	yes	yes
*SRY*				yes	
*Sox9*		sox100B	yes	yes	
*FOG2*			yes	yes	
*AMH*			yes	yes	
*DMRT1*	*dsx*	*MAB-3*	yes	yes	
*DMRT3*			yes	yes	
*DMRT6*				yes	
*DHH*	yes		yes	yes	
*MAP3K1*			yes	yes	yes
*Map3k4*			yes	yes	yes
*ATRX*	dATRX	xnp-1		yes	yes
*Fgf9*				yes	
*Gadd45g*			yes	yes	yes
*Hhat*	yes	yes	yes	yes	yes
*Kdm3a*	yes			yes	
*Dax1*			yes	yes	yes
*Six1–Six4*	yes	yes	yes	yes	yes
*Sox3*			yes	yes	
*Sox8*				yes	yes
*Sox10*				yes	
GSDF				yes	
PDGF α and β				yes	
*AMHR2*			yes	yes	
AR			yes	yes	
SRD5A1, SRD5A2, SRD5A3			yes	yes	yes
CYP11B			yes	yes	
*WNT4*	yes		yes	yes	yes
*FOXL2*			yes	yes	yes
*RSPO1*				yes	
*β-catenin*	*armadillo*		yes	yes	yes
*FST*			yes	yes	yes
*Cyp19A1*			yes	yes	
*ERa*			yes	yes	yes
*Xol-1*		yes			
*Sdc*	yes	yes	yes	yes	
*Her*	yes	yes			
*Tra*	yes	yes	yes	yes	yes
*Fem*	yes	yes	yes		yes
*Fru*	yes				
*Sis*	yes				
*Run*	yes	yes	yes	yes	yes
*Sxl*	yes				
*Doa*	yes		CLK	CLK	yes

C, *Caenorhabditis elegans* (nematode); D, *Drosophila melanogaster* (fruit fly); Dr, *Danio rerio* (zebrafish); M, *Mus musculus* (mouse);Ch, *Crassostrea hongkongensis* (Oyster)

**Fig 6:**
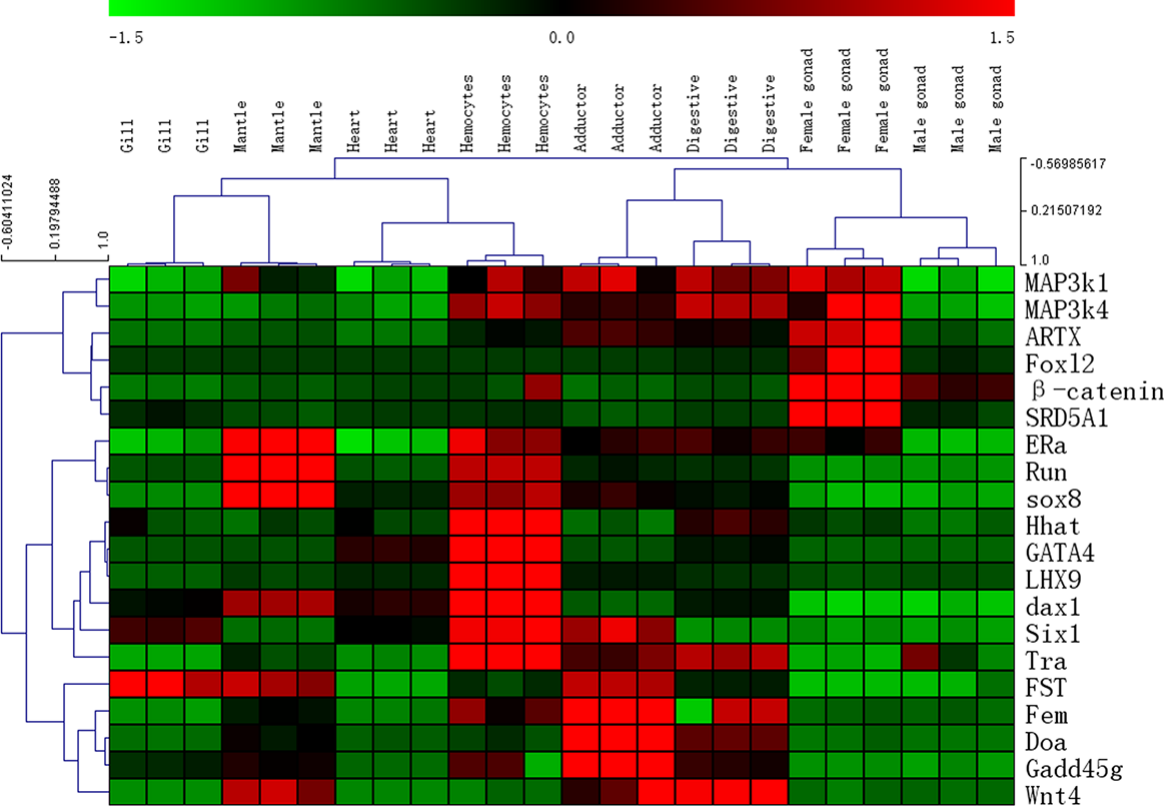
Heat map of sex determination/differentiation genes expressed at different adult tissues of *C*.*hongkongensis*. Genes showing similar expression profiles on all samples (columns) were clustered together. Color represents the normalized expression after variance-stabilizing transformation. Expression levels are depicted with a color scale, in which shades of red represent higher expression and shades of green represent lower expression.

To identify whether these 20 genes function in the *C*.*hongkongensis* reproduction process, we studied the expression profiles of these genes in different tissues by qRT-PCR. We found that 4 genes (ATRX, Foxl2, β-catenin and SRD5A1) were expressed at a higher level in female gonads than in other tissues, with Foxl2 specifically expressed in female gonads, suggesting that they may play important roles in female sex determination/differentiation or maintenance of female mature gonads in *C*.*hongkongensis*. The other 16 genes (Doa, ER a, Fem, FST, GATA4, Gadd45g, Hhat, LHX9, MAP3k1, Map3k4, Dax1, Run, Six1, Sox8, Tra and WNT4) did not show high expression levels in gonads (Fig 6), suggesting that they may not function in the *C*.*hongkongensis* reproduction process. However, we cannot rule out the possibility that they may exhibit high expression levels at earlier stages.

*α-thalassemia/mental retardation syndrome X-linked(ATRX) gene* ATRX is a chromatin remodeling protein that plays a critical and conserved role in mammalian and nematode sexual differentiation [48,49]. Mutations in human *ATRX* cause mental retardation, craniofacial deformities, psychomotor failure, alpha-thalassemia as well as urogenital abnormalities ranging from undescended testes to testicular dysgenesis and female or ambiguous external genitalia [50]. In *C*. *elegans*, the *ATRX* ortholog (*xnp-1*) acts in concert with *lin35* (the ortholog to mammalian retinoblastoma protein) to regulate target gene expression. Knockout of either *xnp-1* or *lin35* alone does not produce a phenotype. However, the double mutants were sterile with severe defects in gonadal development and a decrease in germ cell numbers in both male and female individuals [49]. The human ATRX contains an ADD (ATRX, DNMT3b and DNMT3L) zinc finger domain and a Sucrose Non Fermenting 2 (SNF2)-like DNA-dependent ATPase domain [51,52]. The ADD domain is composed of two zinc fingers (PHD-like and GATA-like zinc fingers) that bind to histone tails and mediate ATRX binding to chromatin. However, in *C*.*elegans* and *D*.*melanogaster*, the putative ATRX homolog only has a conserved SNF2-like DNA-dependent ATPase domain and lack the ADD domain [53,54]. However, little is known about *ATRX* in mollusks.

**Fig 7:**
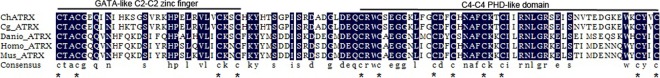
Amino acid sequence alignment of the ADD domain from *C*.*hongkongensis ATRX* ortholog with vertebrate ATRX. The highly conserved C2-C2 zinc finger and C4-C4 zinc finger are indicated by asterisks.

A sequence of 6,767 bp (isotig16330) encoding an *ATRX* ortholog was identified, showing an ORF of 5,607 bp. The deduced amino acid sequence is 1,868 aa long and contains an ADD domain and a SNF2-like DNA-dependent ATPase domain, suggesting that *ATRX* ortholog in *C*.*hongkongensis* has the same structural feature as mammalian ATRX rather than *C*.*elegans* and *D*.*melanogaster* ATRX homologs. This *C*.*hongkongensis* ATRX ortholog mRNA was named “*Ch-ATRX*”. Alignment of the amino acid sequence of the ADD domain from oyster *ATRX* ortholog with vertebrate ATRX indicated high conservation of a C2-C2 zinc finger and a C4-C4 zinc finger (Fig 7). *Ch-ATRX* is highly expressed in female gonads with relatively low expression levels in other organs. In mammals, *ATRX* was strongly expressed in both female and male gonads [48], but in our investigation, we observed that *ChATRX* exhibits higher mRNA levels in the ovary than in the testis, suggesting that *ChATRX* may play a more important role in ovary development in *C*.*hongkongensis*. The role of *ATRX* in the molluscan ovary remains unknown and awaits further investigation.

β-catenin is a subunit of the cadherin protein complex and acts as an intracellular signal transducer in the Wnt signaling pathway. *Wnt4* and *Rspo1*, two components of the Wnt signaling pathway, activate β-Catenin, which in turn regulates the transcription of a variety of genes, among them important ovarian components, such as *Wnt4* and *Fst* [55,56]. The expression of the stabilized form of β-Catenin in the developing mouse XY gonad leads to male-to-female sex-reversal [55]. In mollusks, a *β-catenin* ortholog has been identified in the Pacific oyster *C*. *gigas* (*Cg-β-catenin*). *Cg-β-catenin* is strongly expressed in mature female gonads with low expression levels in male gonads [57].

A 3,888 bp transcript encoding a *β-catenin*ortholog (isotig11203) was identified in *C*.*hongkongensis* transcriptome, having an ORF of 2,499 bp in length. The deduced amino acid sequence is 832 aa long and contains an armadillo repeat region, which is a common feature of β-catenin in other species. Phylogenetic analysis based on the amino acid sequences of β-catenin from various species indicate that the *C*.*hongkongensis β-catenin* ortholog was closely related to *Cg-β-catenin* and was clustered into the molluscan *β-catenin* group (Figure A in S2 Fig). Therefore, we named this *C*.*hongkongensis β-catenin* ortholog (isotig11203) “*Ch-β-catenin*”. *Ch-β-catenin* mRNA expression, measured by real-time quantitative RT-PCR, was detected in all tested adult tissues, but was maximal in female gonads. The expression pattern of *Ch-β-catenin* was similar to that of *Cg-β-catenin* [57], suggesting a more important role of *β-catenin* in oyster female sex differentiation rather than male sex differentiation.

*Forkhead box L2-related gene* The forkhead box L2 gene (*FoxL2*), which encodes a winged helix/forkhead transcription factor, is a key gene in ovarian determination in vertebrates [58]. In adult mammals, FoxL2 is mainly expressed in the ovary where it functions to suppress genes involved in testis differentiation from early embryonic gonad differentiation throughout adult life [59,60,61]. Homologues of *FoxL2* have also been reported in invertebrates [62,63,64]. In mollusks, a FoxL2 ortholog has been identified in *C*.*gigas* (*CgFoxL2*). *CgFoxL2* is expressed in labial palps, female and male gonads, with significantly higher expression levels in female gonads [7,63].

In *C*.*hongkongensis* transcriptome, a 662 bp transcript (isotig29919) related to *FoxL2* were identified by BlastX against the Swiss-prot and Nr database. The ORF is 641 bp encoding a 215 aa product that harbors a forkhead box domain. In phylogenetic analysis using amino acid sequences of FoxL1 and FoxL2 from various species, the product of isotig29919 was aligned to the clade of FoxL2 proteins and was closely related to *CgFoxL2* (Figure B in S2 Fig). Therefore, we named isotig29919 “*ChFoxL2*”. *ChFoxL2* was specifically expressed in ovary, suggesting a key role in ovary development. It will be very interesting to study the regulatory role of *ChFoxL2* in female sex determination/differentiation of *C*.*hongkongensis* in the future.

*5a-reductase1 gene* (*SRD5A1*) 5a-reductase 1 enzyme, encoded by the *SRD5A1* gene, has two important physiological functions in model animals: (i) catalyze the conversion of testosterone into a more potent androgen, dihydrotestosterone (DHT), which participates in the sexual differentiation processes [65]; and (ii) convert progesterone and deoxycorticosterone (DOC) to the irrespective 5-reduced derivatives, precursors of allopregnanolone and tetrahydroDOC, potent allosteric modulators of the γ-aminobutyricacid receptor(GABA_A_-R) [66], which participates in the regulation of various psychophysiological phenomena [67,68]. Homologues of *SRD5A1* have been reported in human [69], rat [70], bird [71], fish [72] and frog [73]. The expression pattern of *SRD5A1* was different among the different species. In the adult rat, *SRD5A1*was expressed at high levels in non androgen target tissues (e.g., liver, brain, ovary and skin) and at lower levels in theprostate, epididymis, seminal vesicles, testis [65,70]. In *Xenopus laevis*, *SRD5A1* mRNA was expressed in all examined tissues with the highest expression in brain, gonads and kidney and lower expression in liver, heart and spleen [74].To date, there have been few reports on the expression pattern of *SRD5A1* in mollusks and its role in molluscan reproduction.

In the transcriptome, we identified a transcript (isotig26744) that encodes 5a-reductase 1 enzyme (*SRD5A1*) with strong similarity to mouse *SRD5A1* (E-value = 1.34E-82). The transcript was 897 bp long with an ORF of 804 bp, which encoded a protein of 267 amino acids. In phylogenetic analysis based on amino acid sequences of SRD5A1 orthologs, the product encoded by isotig26744 was clustered into the SRD5A1 group and was most closely related to *C*.*gigasSRD5A* (Figure C in S2 Fig). Therefore, the isotig26744 was identified for *Ch-SRD5A1*. *Ch*-*SRD5A1* mRNA was expressed highly in female gonad. The high expression in the ovary supports a possible role of *Ch*-*SRD5A1* in determining or promoting female-specific development.

### Oocyte maturation pathway genes

The mechanism underlying oocyte maturation in oysters is poorly understood. In oyster *C*.*gigas* and *C*.*hongkongensis*, oocytes are naturally arrested at the prophase of meiosis I, undergo meiosis re-initiation and germinal vesicle breakdown (GVBD) upon environmental stimuli or hormonal stimulation, are secondarily arrested in metaphase I and become mature, fertilizable eggs [75]. Here, 41 genes of the oocyte maturation pathway, which include MAPK pathway genes, CDC2, cyclin B, polo-like kinase and CDC25 etc., were found in the *C*.*hongkongensis* transcriptome (S3 Fig). Stephano and Gould found that oocytes immediately after removal from the *C*.*gigas* ovary exhibit little MAPK activity, but this increases as the oocytes mature to metaphase I. When MAPK activation is inhibited, meiosis is abnormal, suggesting that MAPK plays an important role in *C*.*gigas* oocyte maturation [76]. In model organisms such as *Xenopus*, the entry into meiosis I depends on the activation of maturation promoting factor (MPF or Cdc2/cyclin B), which triggers GVBD. The MAPK pathway and the polo-like kinase/CDC25 pathway are responsible for the activation of MPF in meiosis [77]. However, in *C*.*hongkongensis*, further investigations are needed to determine the functional roles of genes identified here in the oocyte maturation process.

Overall, our results show that 454 sequencing of the *C*.*hongkongensis* transcriptome was useful in identifying reproduction related genes because a large number of reproduction-related genes and pathways have been identified in *C*.*hongkongensis*.

### Characterization of SNPs and SSRs

Molecular markers such as SNPs or SSRs are the basis for genetic mapping and comparative genomic analysis, which are in turn used for the detection of quantitative trait loci (QTL) and for marker assisted selection (MAS) programs [78]. Although some microsatellite markers have been developed in *C*.*hongkongensis* [79,80,81], few studies have been conducted to investigate cDNA associated SNPs and SSRs in this species, despite the potential for targeting candidate genes.

**Fig 8:**
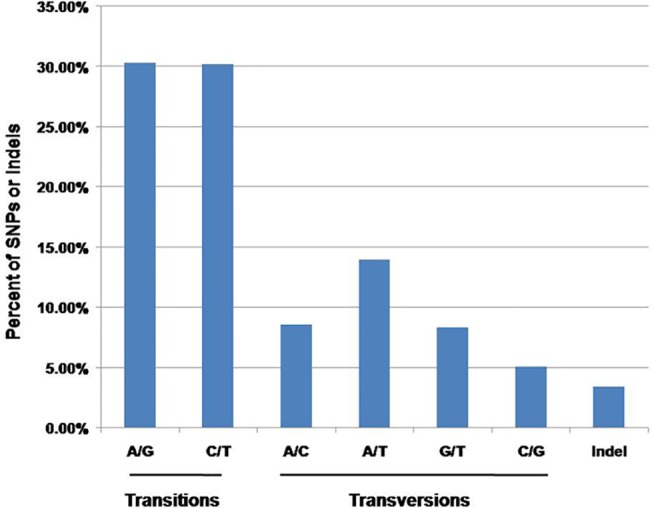
Classification of single nucleotide polymorphisms (SNPs) identified from the *C*.*hongkongensis* transcriptome. Transitions occurred more frequently than transversions. The overall frequency of all types of SNPs including indels was one per 392 bp.

A total of 94,056 SNPs and 3,357 indels were detected in 11,427 isotigs of the *C*.*hongkongensis* transcriptome. The overall frequency of all SNP types was 1 per 392bp.The proportions of transition substitutions were 30.3% for A/G and 30.2% for C/T, compared with smaller proportions of transversions for A/C (8.6%), G/T (8.3%),A/T (14.0%) and C/G (5.1%; Fig 8).

In addition, our search revealed 1,452 isotigs containing 1,699 SSRs in the 22,829 isotigs, with 208 isotigs containing at least two SSRs. Of these, 789 showed significant hits in BlastX against the Swiss-prot database with an E-value cutoff of ≤ 1e-6 and were thus annotated. The frequency of EST-SSRs observed in the transcriptome was 6.4%, and the distribution density was one SSR per 21.7 kb of expressed sequences. The most abundant repeat type was AG (30.2%, 513) followed by AT (20.9%, 356), AC (11.4%, 194) and ATC (0.09%, 157). Regarding the length of the motif, dinucleotide microsatellites were the most common ones and hexanucleotides were the least abundant (Table 5). In addition, SSRs with a lower number of repeats were more common than those with a higher number of repeats, with the most common class being n = 6 (579 loci: 34.1%). Furthermore, 14.2% of loci contained more than 10 repeat units.

**Table 5:** Frequency distribution of SSRs by motif length in the *C*.*hongkongensis* transcriptome.

SSR motif length	Repeat unit number								
	5	6	7	8	9	10	>10	Total	%
Di	-	439	187	107	62	43	225	1,063	62.5%
Tri	331	119	39	28	16	10	10	553	32.5%
Tetra	32	17	10	3	0	0	7	69	4.1%
Penta	7	4	1	1	0	0	0	13	0.07%
Hexa	1	0	0	0	0	0	0	1	0.005%
Total	371	579	237	139	78	53	242	1699	100%

We next searched the seven genes (*Chvasa*, *Ch-nanos*, *ChPiwil1*, *Ch-ATRX*, *Ch-β-catenin*, *ChFoxL2*, *Ch-SRD5A1*) which were expressed highly in *C*.*hongkongensis* gonad for possible SNPs and SSRs. 17 SNPs were identified in *Ch-ATRX*, followed by 9 in *Chvasa*, 7 in *Ch-nanos*, 2 in *Ch-SRD5A1* and 1 in *Ch-β-catenin* (S9 Table). No SNPs was detected in *ChPiwil1* and *ChFoxL2*. One TCC type SSR was identified in *Chvasa* and no SSRs was identified in other genes.

The large number of potential molecular markers found in this study will be useful for gene mapping in this species and for comparative mapping and oyster evolutionary studies.

## Conclusions

In conclusion, our study provided the first assembled transcriptome for *C*. *hongkongensis*, an economically important shellfish in South China. Based on GO, KEGG classification and literature-based searches for known reproduction-related genes, we conclude that our study captured a significant number of genes that may be involved in reproduction. The group of reproduction-related genes identified here constitutes a new tool for research on the bivalve reproduction process and provided insights into the origin and ancient characteristics of the reproduction-related genes. The large set of molecular markers discovered here will be useful for population studies and marker-assisted selection programs in *C*.*hongkongensis* aquaculture.

## Supporting Information

S1 Fig Size distribution of assembled contigs and isotigs. (PPTX)

S2 Fig Molecular phylogenetic tree of β-catenin, FoxL2, SRD5A1 and related proteins. (PPTX)

S3 Fig Progesterone-mediated oocyte maturation pathway representing the present (in green) and absent genes (without color) in the *C*.*hongkongensis* transcriptome. (PPTX)

S1 Table Sample preparation for 454 transcriptome sequencing. (DOCX)

S2 Table Primers used in qRT-PCR and mRNA *in situ* hybridization. (DOCX)

S3 Table Genebank accession numbers of the reference sequences used for sequence analysis. (XLSX)

S4 Table Sequences with significant BLAST matches against Swiss-Prot and NCBI Nt database. (XLSX)

S5 Table The hierarchical list of annotated genes, which is categorized according to the BRITE database. (XLSX)

S6 Table Gene Ontology analysis of the “Reproduction”, “Germline development”, “Sex determination” and “Sex differentiation” terms. (XLSX)

S7 Table Presence of germline development pathway genes from *Drosophila melanogaster*, *Caenorhabditis elegans*, *Danio rerio*, *Mus musculus*. (DOCX)

S8 Table Presence of sex determination/differentiation genes from *Caenorhabditis elegans*, *Drosophila melanogaster*, *Danio rerio*, *Mus musculus* and *Crassostrea hongkongensis*. (DOCX)

S9 Table SNPs and SSRs detected on *Chvasa*, *Ch-nanos*, *Ch-ATRX*, *Ch-β-catenin* and *Ch-SRD5A1*
(XLSX)
