# Supplementary material for: Transcriptomics Analysis of Crassostrea hongkongensis for the Discovery of Reproduction-Related Genes
Source: PLoS One. 2015 Aug 10;10(8):e0134280. doi: 10.1371/journal.pone.0134280 (PMC4530894; doi:10.1371/journal.pone.0134280)
Supplement: S1 Fig — (PPTX) [file pone.0134280.s001.pptx]

## Slide 1
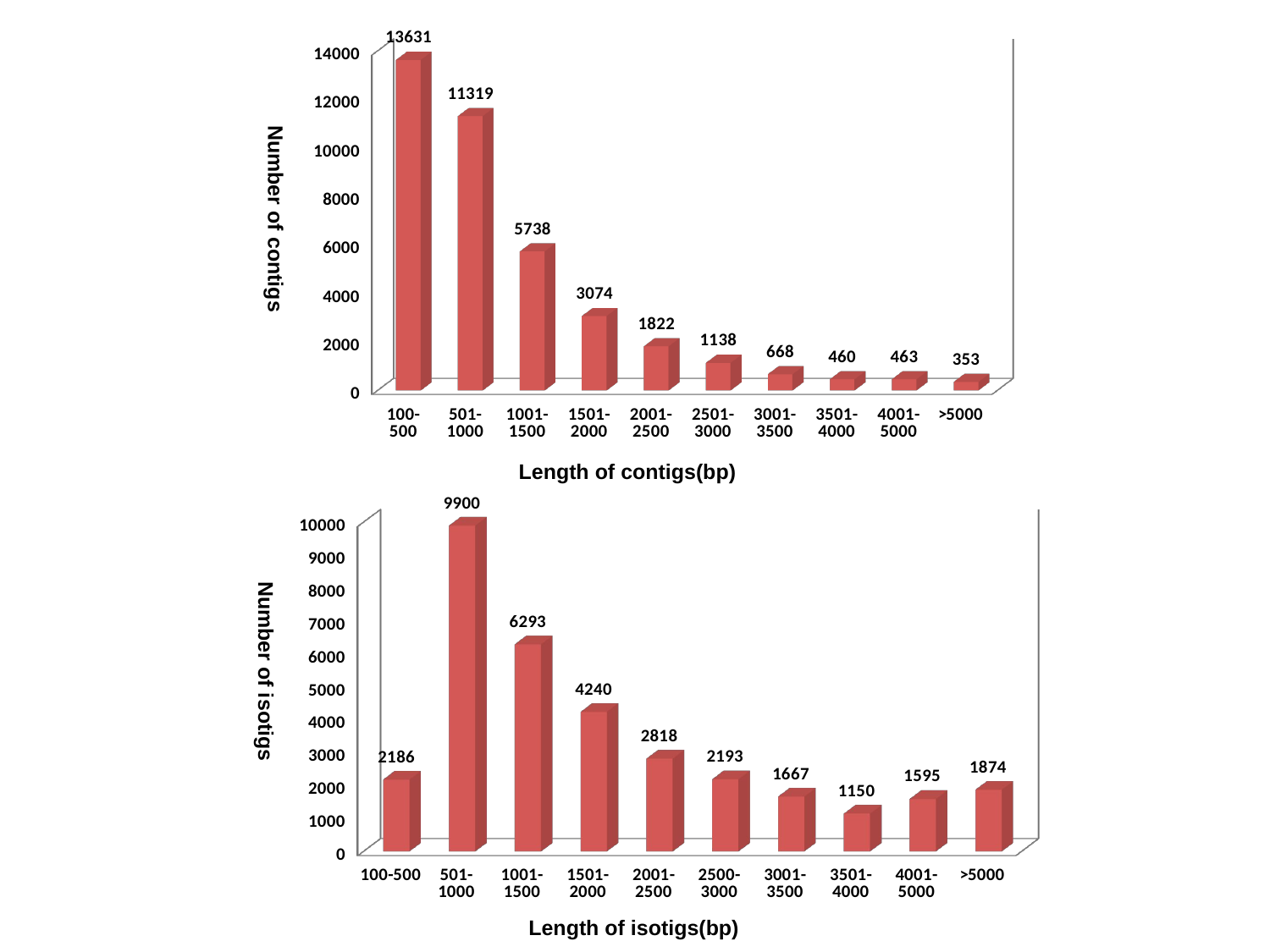

[unsupported chart]
Number of contigs
Length of contigs(bp)
[unsupported chart]
Number of isotigs
Length of isotigs(bp)
