# Supplementary material for: Transcriptomics Analysis of Crassostrea hongkongensis for the Discovery of Reproduction-Related Genes
Source: PLoS One. 2015 Aug 10;10(8):e0134280. doi: 10.1371/journal.pone.0134280 (PMC4530894; doi:10.1371/journal.pone.0134280)
Supplement: S2 Fig — (PPTX) [file pone.0134280.s002.pptx]

## Slide 1
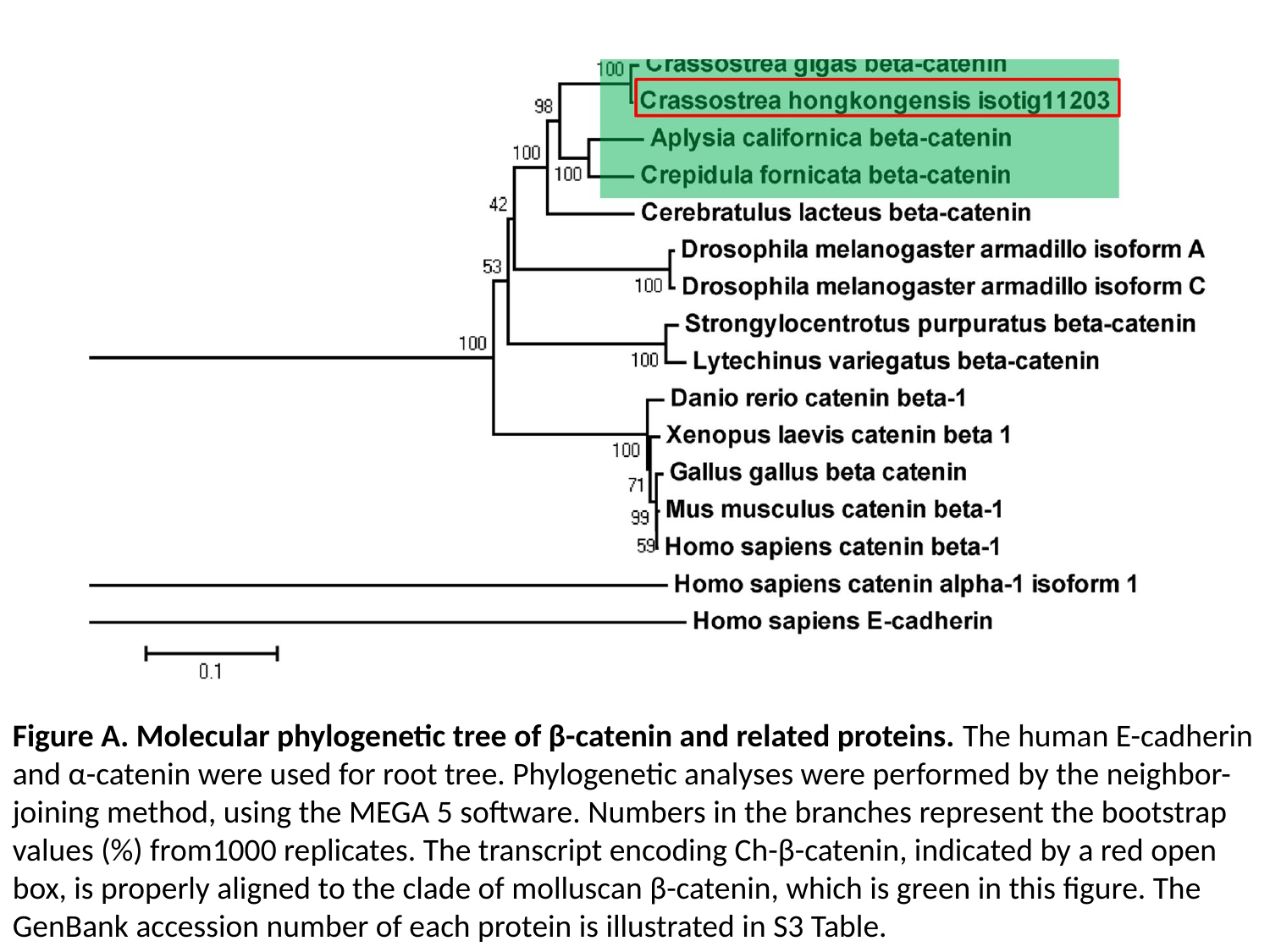

Figure A. Molecular phylogenetic tree of β-catenin and related proteins. The human E-cadherin and α-catenin were used for root tree. Phylogenetic analyses were performed by the neighbor-joining method, using the MEGA 5 software. Numbers in the branches represent the bootstrap values (%) from1000 replicates. The transcript encoding Ch-β-catenin, indicated by a red open box, is properly aligned to the clade of molluscan β-catenin, which is green in this figure. The GenBank accession number of each protein is illustrated in S3 Table.

## Slide 2
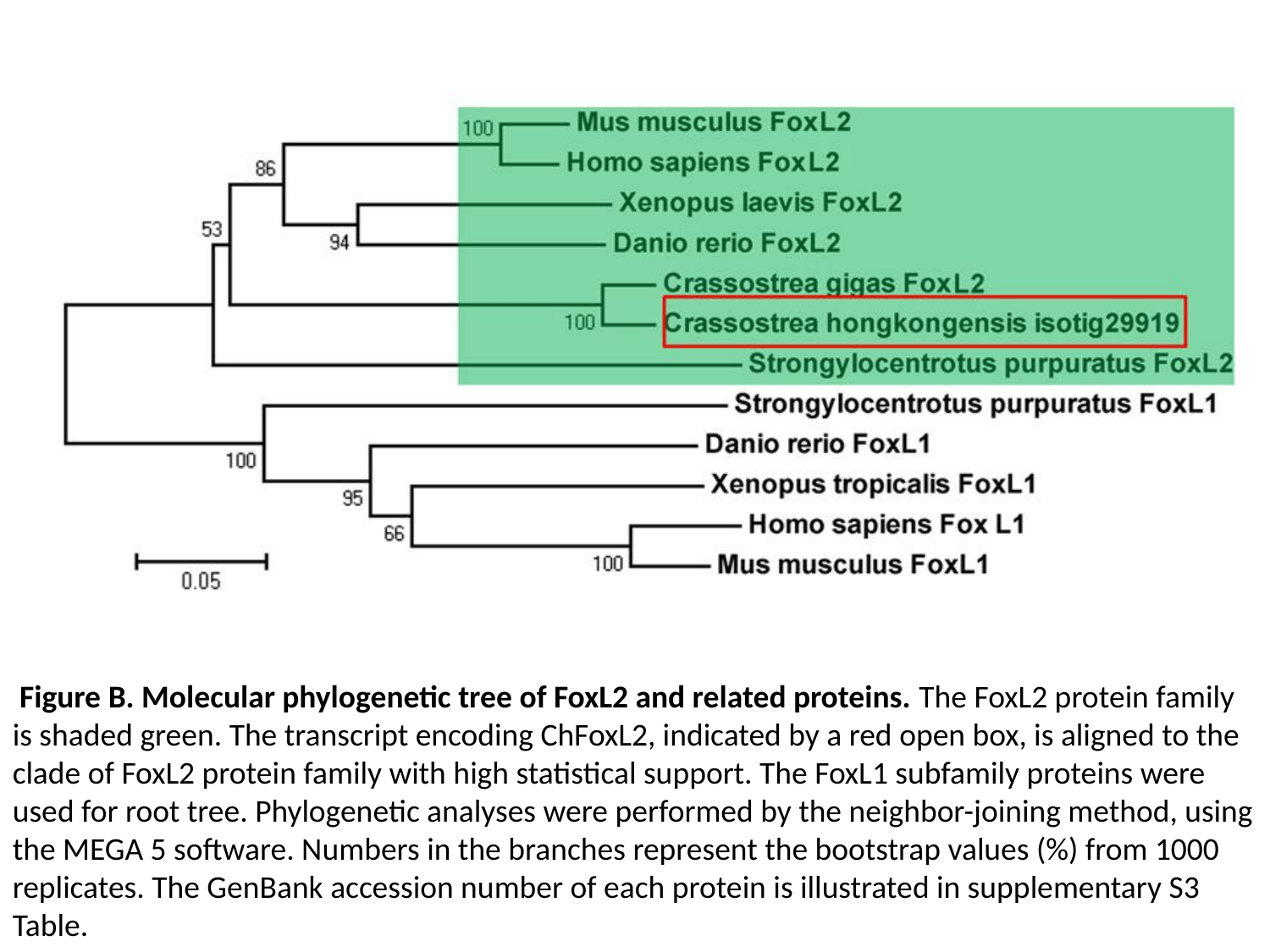

Figure B. Molecular phylogenetic tree of FoxL2 and related proteins. The FoxL2 protein family is shaded green. The transcript encoding ChFoxL2, indicated by a red open box, is aligned to the clade of FoxL2 protein family with high statistical support. The FoxL1 subfamily proteins were used for root tree. Phylogenetic analyses were performed by the neighbor-joining method, using the MEGA 5 software. Numbers in the branches represent the bootstrap values (%) from 1000 replicates. The GenBank accession number of each protein is illustrated in supplementary S3 Table.

## Slide 3
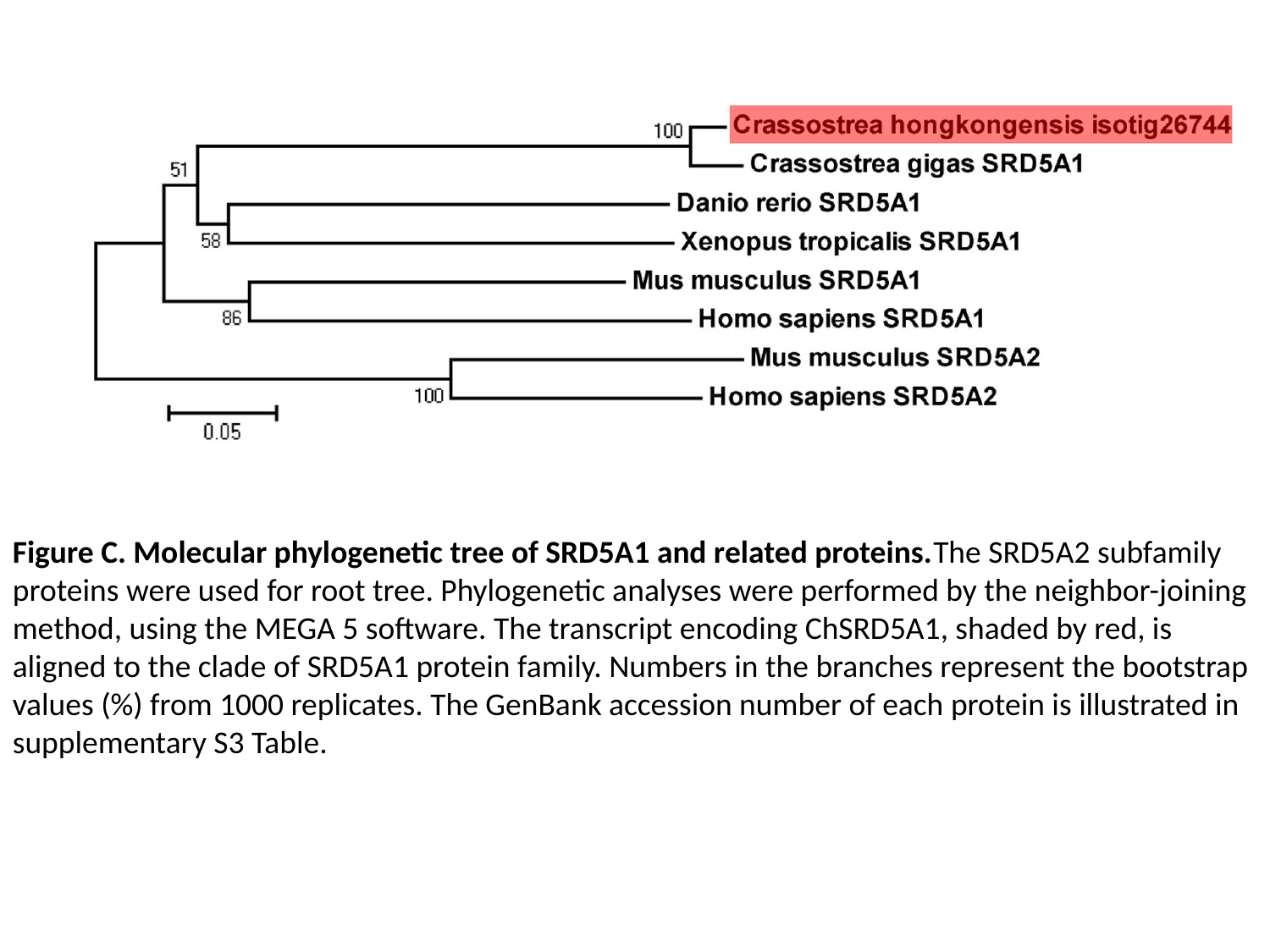

Figure C. Molecular phylogenetic tree of SRD5A1 and related proteins.The SRD5A2 subfamily proteins were used for root tree. Phylogenetic analyses were performed by the neighbor-joining method, using the MEGA 5 software. The transcript encoding ChSRD5A1, shaded by red, is aligned to the clade of SRD5A1 protein family. Numbers in the branches represent the bootstrap values (%) from 1000 replicates. The GenBank accession number of each protein is illustrated in supplementary S3 Table.
