# Supplementary figures and images for: Transcriptomics Analysis of Crassostrea hongkongensis for the Discovery of Reproduction-Related Genes
Source: PLoS One. 2015 Aug 10;10(8):e0134280. doi: 10.1371/journal.pone.0134280 (PMC4530894; doi:10.1371/journal.pone.0134280)

## Slide 1
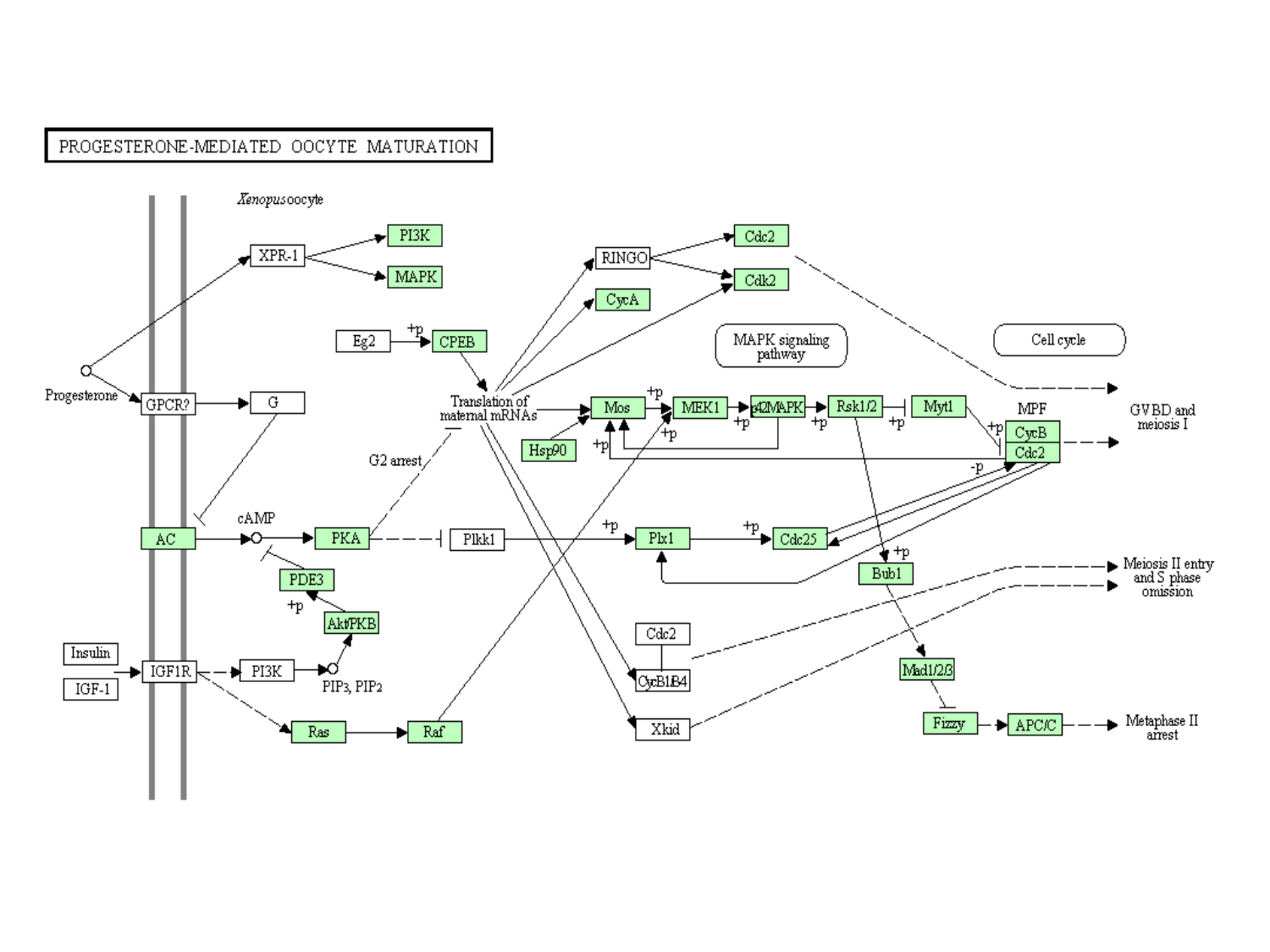

Supplement: S3 Fig — (PPTX) [file pone.0134280.s003.pptx]
