# Supplementary material for: Transcriptomics Analysis of Crassostrea hongkongensis for the Discovery of Reproduction-Related Genes
Source: PLoS One. 2015 Aug 10;10(8):e0134280. doi: 10.1371/journal.pone.0134280 (PMC4530894; doi:10.1371/journal.pone.0134280)
Supplement: S1 Table — (DOCX) [file pone.0134280.s004.docx]

**Table S6 Sample preparation for 454 transcriptome sequencing.** Equal amount of RNA from different somatic tissues, male and female gonads and larvae at different developmental stages were taken out and mixed together to form three RNA samples. Then RNA from each sample was combined into a single pool at different ratios.

| RNA sample | Ratio |
| --- | --- |
| Gill, Adductor muscle, Digestive glands, Hemocytes, Mantle and Heart | 40% |
| Male and female gonad | 20% |
| Larvae of 4-cell stage, Blastula, Trochophore and D-shaped larva stage | 40% |
