# Supplementary material for: Transcriptomics Analysis of Crassostrea hongkongensis for the Discovery of Reproduction-Related Genes
Source: PLoS One. 2015 Aug 10;10(8):e0134280. doi: 10.1371/journal.pone.0134280 (PMC4530894; doi:10.1371/journal.pone.0134280)
Supplement: S2 Table — (DOCX) [file pone.0134280.s005.docx]

**Table S7 Primers used in qRT-PCR and mRNA *in situ* hybridization**

| Gene | Primer name and sequence (5’→3’) | Product | Tm | Usage |
| --- | --- | --- | --- | --- |
| EF1α | ch-ef1a-qsense: GCCCTGTGATGGGTCACTCTTCT  ch-ef1a-qanti: TGGTTCCCAGCAAGCCTATGTG | 137bp | 58℃ | qRT-PCR |
| Vasa | ch-vasa-qsense: ACTAAGATGTGGGTTCCCTGTTC  ch-vasa-qanti: GCAGTAGTTTCTCCGAGGTCC | 190bp | 58℃ | qRT-PCR |
| Nanos | ch-nanos-qsense:ACCAGGTAGAACTTCGGAGACA  ch-nanos-qanti: GGCTTGAGTAGATGGCAACAGT | 174bp | 58℃ | qRT-PCR |
| Piwl2 | ch-piwl2-qsense: CCAACAGGAAAGACCGATACG  ch-piwl2-qanti: GAATGTCCAGGCACCAGACC | 180bp | 58℃ | qRT-PCR |
| *ATRX* | ch-artx-qsense: TAACAATGGCTTTCTGGAACTCG  ch-artx-qanti: CGCAGCAAGACAACAAACCTAA | 111bp | 58℃ | qRT-PCR |
| *Ctnnb1/ β-catenin* | ch-cntb-qsense: CCTGCAAACCATGCTCCACT  ch-cntb-qanti: GCGAACACCATCAACATACCCT | 141bp | 58℃ | qRT-PCR |
| *Doa* | ch-doa-qsense: TTGATATTGCGCTCATCTCGT  ch-doa-qanti: TTGCTCTGAAGCTGTAAACTTGC | 149bp | 58℃ | qRT-PCR |
| *ER a* | ch-era-qsense: TTCCTTCCTCTGGCTCCCTT  ch-era-qanti: CAATACCTGCACTATTGACAAACAC | 103bp | 58℃ | qRT-PCR |
| *Fem* | ch-fem-sense: ATTCCCCAGTGCCCAAACC  ch-fem-anti: GGCCCCTCGATACCTTATGTAGTA | 197bp | 58℃ | qRT-PCR |
| *FST* | ch-fst-qsense: TGTCGAGCTAATGCTACCTGTTT  ch-fst-qanti: TGTAATCCACTCCGTCTGTTCC | 160bp | 58℃ | qRT-PCR |
| *FOXL2* | ch-foxl2-qsense: AGCTCAAATTGTGGCGAATACT  ch-foxl2-qanti: ACGCCTTACGCTTAGTGGAAT | 104bp | 58℃ | qRT-PCR |
| *GATA4* | ch-gata4-qsense:CCCGTGTAATTTGTAGTAGAGTCCG  ch-gata4-qanti: AGAGTTGGGTTGTCTTGTGCC | 114bp | 58℃ | qRT-PCR |
| *Gadd45g* | ch-gadd45g-qsense: ATGGTCGGTCTTTTCTGGAGC  ch-gadd45g-qanti: CAGTGGGAGACAATGGAGATGTA | 200bp | 58℃ | qRT-PCR |
| *Hhat* | ch-hhat-qsense: GCCAGCCCATCAGATACAAGA  ch-hhat-qanti: TGGATTGGAACAGATAAGGACG | 190bp | 58℃ | qRT-PCR |
| *LHX9* | ch-lhx9-qsense: GATTCAGAACTCACCTGCTTTGC  ch-lhx9-qanti: TTCGTTCGCCGTGATACCCT | 117bp | 58℃ | qRT-PCR |
| *MAP3K1* | ch-map3k1-qsense: GCTGAATGCGGATGGTAATG  ch-map3k4-qanti: GCAAGATGATGAATGTCCCTGTAA | 158bp | 58℃ | qRT-PCR |
| *Map3k4* | ch-map3k4-qsense: TGACAAAAGGATGGTCCAGTAGT  ch-map3k4-qanti: ATGTCCACGGGAAAGAGGC | 193bp | 58℃ | qRT-PCR |
| *Nr0b1/ Dax1* | ch-dax-qsense: TTCACCGCACCCTTCCCTA  ch-dax-qanti: ACCCGCCATTGTCCTTGTC | 158bp | 58℃ | qRT-PCR |
| *SRD5A1* | ch-srd5a1-qsense: ACGGGATGAATGGGGTAGG  ch-srd5a1-qanti: CTATTACACCGATTGTCCGAAAC | 110bp | 58℃ | qRT-PCR |
| *Run* | ch-run-qsense: ACGTCCCACAAAACGCAAAT  ch-run-qanti: CCGCTGGAAACGATGAAAAT | 101bp | 58℃ | qRT-PCR |
| *Six1* | ch-six1-qsense: TGTTGTTGTTAGACCCGTTGC  ch-six2-qanti: AGCCGTGGGAAAATACCGT | 187bp | 58℃ | qRT-PCR |
| *Sox8* | ch-sox8-qsense: CTGGAATCCCCGTCGCTCAT  ch-sox8-qanti: ACAAGAAAGACTACCCAGACTACAAA | 159bp | 58℃ | qRT-PCR |
| *Tra* | ch-tra-qsense: CAGACGCAGACATACTGGAAATAG  ch-tra-qanti: TCGACCAAAGACCTCCCTCA | 112bp | 58℃ | qRT-PCR |
| *WNT4* | ch-wnt4-qsense: CGAGCAATGGCACCTTTCAG  ch-wnt4-qanti: CACAAGCCGTGGATGGTTCT | 105bp | 58℃ | qRT-PCR |
| *Ch-nanos* | Ch-nanos -S: CGAAATGCACGATGGCTCAG  Ch-nanos-A: GCTGCTTTCACTGTCGCTACCTC | 713bp | 55℃ | probe amplification in mRNA in situ hybridization |
| *Chpiwil1* | Chpiwil1-S: TGACGGCCATTGCCCGCTAC  Chpiwil1-A:AAGTGTCTATACCTACCACCATCACATTTT | 1146bp | 55℃ | amplification in mRNA in situ hybridization |
