# Supplementary material for: Transcriptomics Analysis of Crassostrea hongkongensis for the Discovery of Reproduction-Related Genes
Source: PLoS One. 2015 Aug 10;10(8):e0134280. doi: 10.1371/journal.pone.0134280 (PMC4530894; doi:10.1371/journal.pone.0134280)
Supplement: S7 Table — (DOCX) [file pone.0134280.s010.docx]

**Table S4 Presence of germline development pathway genes from *Drosophila melanogaster, Caenorhabditis elegans, Danio rerio* , *Mus musculus***

| **Gene (common name)** | **Species with homologues (homologue names)** | | | | | **Gene product** | **Role in germline development** | **Reference** |
| --- | --- | --- | --- | --- | --- | --- | --- | --- |
|  | Fly (D) | Worm (C) | Fish (Dr) | Mouse (M) | Oyster(Ch) |  |  |  |
| *boule* | yes | yes | yes | yes | yes | RNA binding protein | Meiosis; Germ cell differentiation | [[1](#_ENREF_1), [2](#_ENREF_2)] |
| *B-lymphocyteinducedmaturationpro-*  *tein1* (*blimp1*) | yes | yes | yes | yes | yes | Transcriptional repressor | Formation and speciﬁcation of PGCs | [[3](#_ENREF_3), [4](#_ENREF_4)] |
| *Bone morphogenetic protein-4 (bmp4)* | yes |  | yes | yes | yes | Signalling molecule | Generation of primordial germ cell | [[5](#_ENREF_5)] |
| *Bucky ball*(*buc*) |  |  | yes | yes |  | Novel protein | Germ plasm organization | [[6](#_ENREF_6)] |
| *Aubergine*(*Aub*) | yes |  |  |  |  | Similar to eIFC2 (translation initiation factor) | Translational regulation oskar;  Component of a nanos mRNA localization complex | [[7](#_ENREF_7), [8](#_ENREF_8)] |
| *Bruno*(*Bru*) | yes | yes | yes | yes | yes | RNP-type binding domains | Translational regulation of *osk* and *grk;*  Negatively regulates *gcl* expression | [[9](#_ENREF_9), [10](#_ENREF_10)] |
| *Cappuccino*(*capu*) | yes |  |  |  |  | Actin binding protein | Required for polarity of the egg and embryo | [[11](#_ENREF_11)] |
| *Fragilis* |  |  |  | yes |  | IFN inducible TM family member | Germ cell specification | [[12](#_ENREF_12)] |
| *Germ-cell-less*(*gcl*) | yes | yes | yes | yes | yes | Cell type-specific nuclear pore-associated protein | Germ-cell specification | [[13](#_ENREF_13)] |
| *Germ-line development-1*( *gld-1*) |  | yes |  |  |  | KH motif RNA binding protein | Translational repression | [[14](#_ENREF_14), [15](#_ENREF_15)] |
| *gp130* |  |  |  | yes |  | Cytokine receptor | Mutant has fewer PGCs | [[16](#_ENREF_16)] |
| *Gurken*(*grk*) | yes |  |  |  |  | EGFR ligand | Required early in oogenesis for specification of posterior follicle cell fate and later in oogenesis for dorsal follicle cell fate determination | [[17](#_ENREF_17), [18](#_ENREF_18)] |
| *Gustavus*(*gus*) | yes |  | Spsb1 | yes | Spsb1 | SPRY (sp1A/ryanodine receptor) domain and SOCS (suppressor of cytokine signaling) box-containing(Spsb) family member | Pole plasm localization and germ cells specification | [[19](#_ENREF_19), [20](#_ENREF_20)] |
| *homeless* | yes |  |  |  |  | RNA-dependent ATPase | G plasm component localisation | [[21](#_ENREF_21)] |
| *lin28* | yes | yes | yes | yes | yes | RNA binding protein | PGCs specification | [[22](#_ENREF_22), [23](#_ENREF_23)] |
| *Mago nashi*(*mag*) | yes | yes | yes | yes | yes | Novel protein | Germ plasm assembly;  Germ-line sex determination | [[24](#_ENREF_24), [25](#_ENREF_25)] |
| *mes-2* |  | yes |  |  |  | Similar to *Drosophila*  Enhancer of zeste [E(z)]; | Essential for viability of the germline | [[26](#_ENREF_26), [27](#_ENREF_27)] |
| *mes-3* |  | yes |  |  |  |  | MES-2 and MES-6 localisation | [[26](#_ENREF_26)] |
| *mes-4* |  | yes |  |  |  | Novel protein | Germ cells survival | [[26](#_ENREF_26)] |
| *mes-6* |  | yes |  |  |  | Novel protein | Germ cells survival | [[26](#_ENREF_26)] |
| *mex-1* |  | yes |  |  |  | Zinc finger protein | Restriction of PIE-1 expression and activity to the germline | [[28](#_ENREF_28)] |
| *mex-3* |  | yes |  | yes | yes | RNA-binding protein | Promotion of germline stem cells meiosis | [[29](#_ENREF_29), [30](#_ENREF_30)] |
| *nanos* | yes | yes | yes | yes | yes | CCHC Zinc finger protein | Translational and transcriptional repression; PGCs development | [[31-33](#_ENREF_31)] |
| *orb* | yes |  |  |  |  | RNA binding protein | *osk* localisation; regulation of on-site mRNA translation | [[34](#_ENREF_34), [35](#_ENREF_35)] |
| *oskar* | yes |  |  |  |  | Novel protein | Germ plasm organization | [[36](#_ENREF_36)] |
| *par-1* | yes | yes | yes | yes | yes | Ser/Thr kinase | Regulation of cyclin A localization in Drosophila male germline stem cells | [[37](#_ENREF_37)] |
| *pie-1* |  | yes |  |  |  | Zinc finger protein | Transcriptional repression | [[38](#_ENREF_38)] |
| *P-element induced wimpy testis*(*piwi*) | yes | yes | yes | yes | yes | RNA-binding protein | Germline stem cell maintenance; repression of retrotransposons in mammalian testes | [[39](#_ENREF_39)] |
| proliferation of germ cells (*pog*) |  |  |  | yes |  | Plant homeodomain motifs containing protein | PGCs proliferation | [[40](#_ENREF_40)] |
| *Positive regulatory domain I-binding factor* (*Prdm14*) |  |  | yes | yes |  | PR domain-containing transcriptional regulator | Establishment of the germ cell lineage | [[41](#_ENREF_41)] |
| *Pumilio*(*pum*) | yes | yes | yes | yes | yes | PUF-domain containing | Germline stem cell (GSC) maintenance | [[42](#_ENREF_42)] |
| *Spire* | yes |  |  |  |  | Novel protein | *osk* and *stau* localisation in oocyte | [[43](#_ENREF_43)] |
| *Staufen*(*stau*) | yes | yes | yes | yes | yes | RNA-binding proteins | Germ plasm assembly | [[44](#_ENREF_44)] |
| *tudor* | yes |  | yes | yes | yes | Tudor domain containing protein | Germ plasm assembly; *nos* localisation | [[45](#_ENREF_45), [46](#_ENREF_46)] |
| *Valois*(*vls*) | yes |  |  |  |  | Novel protein | *Vasa* localization and *Oskar* protein accumulation | [[47](#_ENREF_47)] |
| *Vasa* | yes | yes | yes | yes | yes | DEAD-box RNA helicase | Germ cell proliferation | [[48](#_ENREF_48)] |
| C, *Caenorhabditis elegans* (nematode); D, *Drosophila melanogaster* (fruit fly); Dr, *Danio rerio* (zebrafish); M, *Mus musculus* (mouse);Ch, *Crassostrea hongkongensis*; | | | | | |  |  |  |

**References**

1. Xu EY, Moore FL, Pera RA: **A gene family required for human germ cell development evolved from an ancient meiotic gene conserved in metazoans**. *Proc Natl Acad Sci U S A* 2001, **98**(13):7414-7419.

2. VanGompel MJ, Xu EY: **A novel requirement in mammalian spermatid differentiation for the DAZ-family protein Boule**. *Hum Mol Genet* 2010, **19**(12):2360-2369.

3. Ancelin K, Lange UC, Hajkova P, Schneider R, Bannister AJ, Kouzarides T, Surani MA: **Blimp1 associates with Prmt5 and directs histone arginine methylation in mouse germ cells**. *Nat Cell Biol* 2006, **8**(6):623-630.

4. Wilm TP, Solnica-Krezel L: **Essential roles of a zebrafish prdm1/blimp1 homolog in embryo patterning and organogenesis**. *Development* 2005, **132**(2):393-404.

5. Lawson KA, Dunn NR, Roelen BAJ, Zeinstra LM, Davis AM, Wright CVE, Korving JPWFM, Hogan BLM: **Bmp4 is required for the generation of primordial germ cells in the mouse embryo**. *Gene Dev* 1999, **13**(4):424-436.

6. Marlow FL, Mullins MC: **Bucky ball functions in Balbiani body assembly and animal-vegetal polarity in the oocyte and follicle cell layer in zebrafish**. *Dev Biol* 2008, **321**(1):40-50.

7. Harris AN, Macdonald PM: **Aubergine encodes a Drosophila polar granule component required for pole cell formation and related to eIF2C**. *Development* 2001, **128**(14):2823-2832.

8. Becalska AN, Kim YR, Belletier NG, Lerit DA, Sinsimer KS, Gavis ER: **Aubergine is a component of a nanos mRNA localization complex**. *Dev Biol* 2011, **349**(1):46-52.

9. Moore J, Han H, Lasko P: **Bruno negatively regulates germ cell-less expression in a BRE-independent manner**. *Mech Dev* 2009, **126**(7):503-516.

10. Webster PJ, Liang L, Berg CA, Lasko P, Macdonald PM: **Translational repressor bruno plays multiple roles in development and is widely conserved**. *Genes Dev* 1997, **11**(19):2510-2521.

11. Emmons S, Phan H, Calley J, Chen W, James B, Manseau L: **Cappuccino, a Drosophila maternal effect gene required for polarity of the egg and embryo, is related to the vertebrate limb deformity locus**. *Genes Dev* 1995, **9**(20):2482-2494.

12. Lange UC, Saitou M, Western PS, Barton SC, Surani MA: **The fragilis interferon-inducible gene family of transmembrane proteins is associated with germ cell specification in mice**. *BMC Dev Biol* 2003, **3**:1.

13. Jongens TA, Ackerman LD, Swedlow JR, Jan LY, Jan YN: **Germ cell-less encodes a cell type-specific nuclear pore-associated protein and functions early in the germ-cell specification pathway of Drosophila**. *Genes Dev* 1994, **8**(18):2123-2136.

14. Lee MH, Schedl T: **Identification of in vivo mRNA targets of GLD-1, a maxi-KH motif containing protein required for C. elegans germ cell development**. *Genes Dev* 2001, **15**(18):2408-2420.

15. Beadell AV, Haag ES: **Evolutionary dynamics of GLD-1-mRNAs complexes in Caenorhabditis nematodes**. *Genome Biol Evol* 2014.

16. Koshimizu U, Taga T, Watanabe M, Saito M, Shirayoshi Y, Kishimoto T, Nakatsuji N: **Functional requirement of gp130-mediated signaling for growth and survival of mouse primordial germ cells in vitro and derivation of embryonic germ (EG) cells**. *Development* 1996, **122**(4):1235-1242.

17. Shmueli A, Cohen-Gazala O, Neuman-Silberberg FS: **Gurken, a TGF-alpha-like protein involved in axis determination in Drosophila, directly binds to the EGF-receptor homolog Egfr**. *Biochem Biophys Res Commun* 2002, **291**(4):732-737.

18. Hawkins NC, Van Buskirk C, Grossniklaus U, Schupbach T: **Post-transcriptional regulation of gurken by encore is required for axis determination in Drosophila**. *Development* 1997, **124**(23):4801-4810.

19. Xing Y, Gosden R, Lasko P, Clarke H: **Murine homologues of the Drosophila gustavus gene are expressed in ovarian granulosa cells**. *Reproduction* 2006, **131**(5):905-915.

20. Gustafson EA, Yajima M, Juliano CE, Wessel GM: **Post-translational regulation by gustavus contributes to selective Vasa protein accumulation in multipotent cells during embryogenesis**. *Dev Biol* 2011, **349**(2):440-450.

21. Gillespie DE, Berg CA: **Homeless Is Required for Rna Localization in Drosophila Oogenesis and Encodes a New Member of the De-H Family of Rna-Dependent Atpases**. *Gene Dev* 1995, **9**(20):2495-2508.

22. Moss EG, Tang L: **Conservation of the heterochronic regulator Lin-28, its developmental expression and microRNA complementary sites**. *Dev Biol* 2003, **258**(2):432-442.

23. West JA, Viswanathan SR, Yabuuchi A, Cunniff K, Takeuchi A, Park IH, Sero JE, Zhu H, Perez-Atayde A, Frazier AL *et al*: **A role for Lin28 in primordial germ-cell development and germ-cell malignancy**. *Nature* 2009, **460**(7257):909-913.

24. Newmark PA, Mohr SE, Gong L, Boswell RE: **mago nashi mediates the posterior follicle cell-to-oocyte signal to organize axis formation in Drosophila**. *Development* 1997, **124**(16):3197-3207.

25. Li W, Boswell R, Wood WB: **mag-1, a homolog of Drosophila mago nashi, regulates hermaphrodite germ-line sex determination in Caenorhabditis elegans**. *Dev Biol* 2000, **218**(2):172-182.

26. Garvin C, Holdeman R, Strome S: **The phenotype of mes-2, mes-3, mes-4 and mes-6, maternal-effect genes required for survival of the germline in Caenorhabditis elegans, is sensitive to chromosome dosage**. *Genetics* 1998, **148**(1):167-185.

27. Holdeman R, Nehrt S, Strome S: **MES-2, a maternal protein essential for viability of the germline in Caenorhabditis elegans, is homologous to a Drosophila Polycomb group protein**. *Development* 1998, **125**(13):2457-2467.

28. Guedes S, Priess JR: **The C. elegans MEX-1 protein is present in germline blastomeres and is a P granule component**. *Development* 1997, **124**(3):731-739.

29. Ariz M, Mainpal R, Subramaniam K: **C. elegans RNA-binding proteins PUF-8 and MEX-3 function redundantly to promote germline stem cell mitosis**. *Dev Biol* 2009, **326**(2):295-304.

30. Pereira B, Le Borgne M, Chartier NT, Billaud M, Almeida R: **MEX-3 proteins: recent insights on novel post-transcriptional regulators**. *Trends Biochem Sci* 2013, **38**(10):477-479.

31. Beer RL, Draper BW: **nanos3 maintains germline stem cells and expression of the conserved germline stem cell gene nanos2 in the zebrafish ovary**. *Dev Biol* 2013, **374**(2):308-318.

32. Forbes A, Lehmann R: **Nanos and Pumilio have critical roles in the development and function of Drosophila germline stem cells**. *Development* 1998, **125**(4):679-690.

33. Hayashi Y, Hayashi M, Kobayashi S: **Nanos suppresses somatic cell fate in Drosophila germ line**. *Proc Natl Acad Sci U S A* 2004, **101**(28):10338-10342.

34. Castagnetti S, Ephrussi A: **Orb and a long poly(A) tail are required for efficient oskar translation at the posterior pole of the Drosophila oocyte**. *Development* 2003, **130**(5):835-843.

35. Costa A, Pazman C, Sinsimer KS, Wong LC, McLeod I, Yates J, 3rd, Haynes S, Schedl P: **Rasputin functions as a positive regulator of orb in Drosophila oogenesis**. *Plos One* 2013, **8**(9):e72864.

36. Ephrussi A, Dickinson LK, Lehmann R: **Oskar organizes the germ plasm and directs localization of the posterior determinant nanos**. *Cell* 1991, **66**(1):37-50.

37. Yuan H, Chiang CY, Cheng J, Salzmann V, Yamashita YM: **Regulation of cyclin A localization downstream of Par-1 function is critical for the centrosome orientation checkpoint in Drosophila male germline stem cells**. *Dev Biol* 2012, **361**(1):57-67.

38. Mello CC, Schubert C, Draper B, Zhang W, Lobel R, Priess JR: **The PIE-1 protein and germline specification in C. elegans embryos**. *Nature* 1996, **382**(6593):710-712.

39. Aravin AA, Hannon GJ, Brennecke J: **The Piwi-piRNA pathway provides an adaptive defense in the transposon arms race**. *Science* 2007, **318**(5851):761-764.

40. Agoulnik AI, Lu B, Zhu Q, Truong C, Ty MT, Arango N, Chada KK, Bishop CE: **A novel gene, Pog, is necessary for primordial germ cell proliferation in the mouse and underlies the germ cell deficient mutation, gcd**. *Hum Mol Genet* 2002, **11**(24):3047-3053.

41. Yamaji M, Seki Y, Kurimoto K, Yabuta Y, Yuasa M, Shigeta M, Yamanaka K, Ohinata Y, Saitou M: **Critical function of Prdm14 for the establishment of the germ cell lineage in mice**. *Nature Genetics* 2008, **40**(8):1016-1022.

42. Parisi M, Lin H: **The Drosophila pumilio gene encodes two functional protein isoforms that play multiple roles in germline development, gonadogenesis, oogenesis and embryogenesis**. *Genetics* 1999, **153**(1):235-250.

43. Clark I, Giniger E, Ruohola-Baker H, Jan LY, Jan YN: **Transient posterior localization of a kinesin fusion protein reflects anteroposterior polarity of the Drosophila oocyte**. *Curr Biol* 1994, **4**(4):289-300.

44. Heraud-Farlow JE, Kiebler MA: **The multifunctional Staufen proteins: conserved roles from neurogenesis to synaptic plasticity**. *Trends Neurosci* 2014, **37**(9):470-479.

45. Chuma S, Hiyoshi M, Yamamoto A, Hosokawa M, Takamune K, Nakatsuji N: **Mouse Tudor Repeat-1 (MTR-1) is a novel component of chromatoid bodies/nuages in male germ cells and forms a complex with snRNPs**. *Mech Dev* 2003, **120**(9):979-990.

46. Wang C, Dickinson LK, Lehmann R: **Genetics of nanos localization in Drosophila**. *Dev Dyn* 1994, **199**(2):103-115.

47. Cavey M, Hijal S, Zhang X, Suter B: **Drosophila valois encodes a divergent WD protein that is required for Vasa localization and Oskar protein accumulation**. *Development* 2005, **132**(3):459-468.

48. Gustafson EA, Wessel GM: **Vasa genes: emerging roles in the germ line and in multipotent cells**. *Bioessays* 2010, **32**(7):626-637.
