# Supplementary material for: Transcriptomics Analysis of Crassostrea hongkongensis for the Discovery of Reproduction-Related Genes
Source: PLoS One. 2015 Aug 10;10(8):e0134280. doi: 10.1371/journal.pone.0134280 (PMC4530894; doi:10.1371/journal.pone.0134280)
Supplement: S8 Table — (DOCX) [file pone.0134280.s011.docx]

**Table S5 Presence of sex determination/differentiation pathway genes from *Drosophila melanogaster, Caenorhabditis elegans, Danio rerio* , *Mus musculus*, *Crassostrea hongkongensis*.**

| **Gene (common name)** | **Species with homologues (homologue names)** | | | | | **Protein function** | **Role in sex determination/differentiation** | **Reference** |
| --- | --- | --- | --- | --- | --- | --- | --- | --- |
|  | **Fly (D)** | **Worm (C)** | **Fish (Dr)** | **Mouse (M)** | **Oyster(Ch)** |  |  |  |
| *Wilms’ tumor suppressor 1* (*WT1*) |  |  | yes | yes |  | Transcription factor | Bipotential gonad development | [[1](#_ENREF_1)] |
| *Nuclear receptor subfamily 5, group A,member 1 (NR5A1), also known as steroidogenic factor 1*(*Sf1*) |  |  | yes | yes |  | Nuclear receptor ; Transcription factor | Bipotential gonad development | [[2](#_ENREF_2)] |
| *LIM homeobox gene 9 (LHX9)* |  |  | yes | yes | yes | Transcription factor | Bipotential gonad development | [[3](#_ENREF_3)] |
| *Empty-spiracles homeobox gene 2 (EMX2)* |  |  | yes | yes |  | Transcription factor | Bipotential gonad development | [[4](#_ENREF_4)] |
| *GATA-binding protein 4 (GATA4)* |  |  | yes | yes | yes | Transcription factor | Bipotential gonad development | [[5](#_ENREF_5)] |
| *Chromobox homolog 2* (*CBX2*) | yes |  | yes | yes |  | Transcription factor | Testis differentiation; required for Sry gene expression | [[6](#_ENREF_6)] |
| *Sex-Determining Region on the Y Chromosome(SRY)* |  |  |  | yes |  | Transcription factor | Testis differentiation | [[7](#_ENREF_7)] |
| *SRY-Related HMG Box 9(Sox9)* |  | sox100B | yes | yes |  | Transcription factor | Testogenesis | [[8](#_ENREF_8)] |
| *Zinc finger protein, FOG family member 2*(*FOG2,also known as ZFPM2*) |  |  | yes | yes |  | Cofactor of Gata4 | Testis determination | [[9](#_ENREF_9)] |
| *Anti-Müllerian Hormone(AMH)* |  |  | yes | yes |  | Hormone | Mammalian male and female sexual differentiation | [[10](#_ENREF_10)] |
| *Doublesex and mab-3-Related Transcription Factor 1(DMRT1)* | *dsx* | *MAB-3* | yes | yes |  | Transcription factor | Sex determination and differentiation in vertebrate, *C.elegans* and *Drosophila* | [[11](#_ENREF_11)] |
| *DMRT6* |  |  |  | yes |  | Transcription factor | Male gonad development | [[12](#_ENREF_12)] |
| *Desert hedgehog (DHH)* | yes |  | yes | yes |  | Signalling molecule | Male gonad development | [[13](#_ENREF_13)] |
| *Mitogen-activated kinase kinase kinase 3*( *MAP3K1*) |  |  | yes | yes | yes | Kinase | Male gonad development | [[14](#_ENREF_14)] |
| *Mitogen-activated kinase kinase kinase 4 (Map3k4)* |  |  | yes | yes | yes | Kinase | Male sex determination | [[15](#_ENREF_15)] |
| *α-thalassemia/mental retardation syndrome X-linked (ATRX)* | *dATRX* | *xnp-1* |  | yes | yes | Chromatin remodeling protein | Sex differentiation | [[16](#_ENREF_16), [17](#_ENREF_17)] |
| *Fibroblast growth factor 9* ( *Fgf9*) |  |  |  | yes |  | Signaling molecule | Male sex determination | [[18](#_ENREF_18)] |
| *Growth arrest and DNA damage-inducible protein γ*(*Gadd45g*) |  |  | yes | yes | yes | Nuclear protein  protein cofactor | Male sex determination | [[19](#_ENREF_19), [20](#_ENREF_20)] |
| *Hedgehog acyl-transferase* ( *Hhat*) | yes | yes | yes | yes | yes | Membrane-bound acyltransferase | Male gonad development | [[21](#_ENREF_21)] |
| *Lysine-specific demethylase 3A* (*Kdm3a*, also known as *Jmjd1a*) | yes |  |  | yes |  | Zinc finger protein  Histone demethylase | Male sex determination | [[22](#_ENREF_22)] |
| *Nuclear receptor subfamily 0, group B, member 1* ( *Nr0b1*, also known as *Dax1*) |  |  | yes | yes | yes | Nuclear receptor | Male sex determination | [[23](#_ENREF_23)] |
| *Six homeobox 1*; *Six homeobox 4* ( *Six1–Six4*) | yes | yes | yes | yes | yes | Transcription factor | Male sex differentiation | [[24](#_ENREF_24)] |
| *Sry-box 3* ( *Sox3*) |  |  | yes | yes |  | Transcription factor | Male gonad development | [[25](#_ENREF_25), [26](#_ENREF_26)] |
| *Sry-box 8* ( *Sox8*) |  |  |  | yes | yes | Transcription factor | Testis differentiation | [[27](#_ENREF_27)] |
| *Sry-box 10* ( *Sox10*) |  |  |  | yes |  | Transcription factor | Testis differentiation | [[28](#_ENREF_28)] |
| *Gonadal soma derived growth factor on the Y chromosome*(*GSDF*) |  |  |  | yes |  | Signalling molecule | Testis differentiation | [[29](#_ENREF_29)] |
| *Platelet-derived growth factors*(*PDGF*) *α* and *β* |  |  |  | yes |  | Growth-regulatory molecules | Male gonad development | [[30](#_ENREF_30)] |
| PDGF receptors |  |  |  | yes |  | Growth factor receptor | Male gonad development | [[30](#_ENREF_30)] |
| *Anti-Müllerian Hormone receptor 2*(*AMHR2*) |  |  | yes | yes |  | Transcription factor  Hormone receptor | Male gonad development | [[31](#_ENREF_31)] |
| *Androgen receptor*(*AR*) |  |  | yes | yes |  | Transcription factor  Hormone receptor | Male sex differentiation | [[32](#_ENREF_32)] |
| *11b-hydroxylase* (*CYP11B*) |  |  | yes | yes |  | Steroid hormone biosynthesis enzyme | Male sex differentiation | [[33](#_ENREF_33)] |
| 5a-reductase1,  2,and 3 (*SRD5A1, SRD5A2, SRD5A3*)] |  |  | yes | yes | yes | Steroid hormone biosynthesis enzyme | Male sex differentiation | [[34](#_ENREF_34)] |
| *Wingless-Type MMTV Integration Site Family, Member 4*(*WNT4*) | yes |  | yes | yes | yes | Signalling molecule | Female sex determination | [[35](#_ENREF_35)] |
| *Forkhead transcription factor* (*FOXL2*) |  |  | yes | yes | yes | Transcription factor | Female sex determination | [[36](#_ENREF_36)] |
| *R-spondin family 1* (*RSPO1*) |  |  |  | yes |  | Signalling molecule | Female sex determination | [[37](#_ENREF_37)] |
| *catenin, β1*(*Ctnnb1,* also known as *β-catenin*) | *armadillo* |  | yes | yes | yes | Transcription factor | Female sex determination | [[38](#_ENREF_38)] |
| *Follistatin*(*FST*) |  |  | yes | yes | yes | Ligand antagonist of activin | Female sex differentiation | [[39](#_ENREF_39), [40](#_ENREF_40)] |
| *Aromatase* (also known as *Cyp19A1* or *P450arom*) |  |  | yes | yes |  | Steroid hormone biosynthesis enzyme | Female sex differentiation | [[41](#_ENREF_41)] |
| *Estrogen receptor a*(*ERa*) |  |  | yes | yes | yes | Transcription factor  Hormone receptor | Female sex differentiation | [[42](#_ENREF_42)] |
| *X0 lethal*(*Xol-1*) |  | yes |  |  |  | Transcription factor | Male sex determination | [[43](#_ENREF_43)] |
| *Sex and dosage compensation*(*Sdc*) | yes | yes | yes | yes |  | Transcription factor | Female sex determination | [[44](#_ENREF_44)] |
| *Her* | yes | yes |  |  |  | Transcription factor | Male sex determination | [[45](#_ENREF_45)] |
| *Transformer*(*Tra*) | yes | yes | yes | yes | yes | Transcription factor | Female sex determination | [[46](#_ENREF_46)] |
| *Fem* | yes | yes | yes |  | yes | Transcription factor | Male sex determination | [[47](#_ENREF_47)] |
| *Fruitless*(*Fru*) | yes |  |  |  |  | Transcription factor | Male sex differentiation | [[48](#_ENREF_48)] |
| *Sisterless*(*Sis*) | yes |  |  |  |  | Transcription factor | Female sex determination | [[49](#_ENREF_49)] |
| *Runt*(*Run*) | yes | yes | yes | yes | yes | Transcription factor | Female sex determination | [[50](#_ENREF_50)] |
| *Sex-lethal* (*Sxl*) | yes |  |  |  |  | Transcription factor | Female sex determination | [[51](#_ENREF_51)] |
| *Darkener of apricot*(*Doa*) | yes |  | *CDC-like kinase*(*CLK*) | *CDC-like kinase*(*CLK*) | yes | Kinase | Female sex determination | [[52](#_ENREF_52)] |
| C, *Caenorhabditis elegans* (nematode); D, *Drosophila melanogaster* (fruit fly); Dr, *Danio rerio* (zebrafish); M, *Mus musculus* (mouse);Ch, *Crassostrea hongkongensis* | | | | | |  |  |  |

**References**

1. Kreidberg JA, Sariola H, Loring JM, Maeda M, Pelletier J, Housman D, Jaenisch R: **Wt-1 Is Required for Early Kidney Development**. *Cell* 1993, **74**(4):679-691.

2. Luo XR, Ikeda YY, Parker KL: **A Cell-Specific Nuclear Receptor Is Essential for Adrenal and Gonadal Development and Sexual-Differentiation**. *Cell* 1994, **77**(4):481-490.

3. Birk OS, Casiano DE, Wassif CA, Cogliati T, Zhao LP, Zhao YG, Grinberg A, Huang SP, Kreidberg JA, Parker KL *et al*: **The LIM homeobox gene Lhx9 is essential for mouse gonad formation**. *Nature* 2000, **403**(6772):909-913.

4. Miyamoto N, Yoshida M, Kuratani S, Matsuo I, Aizawa S: **Defects of urogenital development in mice lacking Emx2**. *Development* 1997, **124**(9):1653-1664.

5. Hu YC, Okumura LM, Page DC: **Gata4 is required for formation of the genital ridge in mice**. *PLoS Genet* 2013, **9**(7):e1003629.

6. Katoh-Fukui Y, Miyabayashi K, Komatsu T, Owaki A, Baba T, Shima Y, Kidokoro T, Kanai Y, Schedl A, Wilhelm D *et al*: **Cbx2, a Polycomb Group Gene, Is Required for Sry Gene Expression in Mice**. *Endocrinology* 2012, **153**(2):913-924.

7. Koopman P, Gubbay J, Vivian N, Goodfellow P, Lovell-Badge R: **Male development of chromosomally female mice transgenic for Sry**. *Nature* 1991, **351**(6322):117-121.

8. Barrionuevo F, Bagheri-Fam S, Klattig J, Kist R, Taketo MM, Englert C, Scherer G: **Homozygous inactivation of Sox9 causes complete XY sex reversal in mice**. *Biol Reprod* 2006, **74**(1):195-201.

9. Bouma GJ, Washburn LL, Albrecht KH, Eicher EM: **Correct dosage of Fog2 and Gata4 transcription factors is critical for fetal testis development in mice**. *P Natl Acad Sci USA* 2007, **104**(38):14994-14999.

10. Munsterberg A, Lovell-Badge R: **Expression of the mouse anti-mullerian hormone gene suggests a role in both male and female sexual differentiation**. *Development* 1991, **113**(2):613-624.

11. Kopp A: **Dmrt genes in the development and evolution of sexual dimorphism**. *Trends Genet* 2012, **28**(4):175-184.

12. Zhang T, Murphy MW, Gearhart MD, Bardwell VJ, Zarkower D: **The mammalian Doublesex homolog DMRT6 coordinates the transition between mitotic and meiotic developmental programs during spermatogenesis**. *Development* 2014, **141**(19):3662-3671.

13. Bitgood MJ, Shen L, McMahon AP: **Sertoli cell signaling by Desert hedgehog regulates the male germline**. *Curr Biol* 1996, **6**(3):298-304.

14. Warr N, Bogani D, Siggers P, Brixey R, Tateossian H, Dopplapudi A, Wells S, Cheeseman M, Xia Y, Ostrer H *et al*: **Minor abnormalities of testis development in mice lacking the gene encoding the MAPK signalling component, MAP3K1**. *Plos One* 2011, **6**(5):e19572.

15. Bogani D, Siggers P, Brixey R, Warr N, Beddow S, Edwards J, Williams D, Wilhelm D, Koopman P, Flavell RA *et al*: **Loss of mitogen-activated protein kinase kinase kinase 4 (MAP3K4) reveals a requirement for MAPK signalling in mouse sex determination**. *PLoS Biol* 2009, **7**(9):e1000196.

16. Huyhn K, Renfree MB, Graves JA, Pask AJ: **ATRX has a critical and conserved role in mammalian sexual differentiation**. *BMC Dev Biol* 2011, **11**:39.

17. Lee NG, Hong YK, Yu SY, Han SY, Geum D, Cho KS: **dXNP, a Drosophila homolog of XNP/ATRX, induces apoptosis via Jun-N-terminal kinase activation**. *FEBS Lett* 2007, **581**(14):2625-2632.

18. Kim Y, Kobayashi A, Sekido R, DiNapoli L, Brennan J, Chaboissier MC, Poulat F, Behringer RR, Lovell-Badge R, Capel B: **Fgf9 and Wnt4 act as antagonistic signals to regulate mammalian sex determination**. *Plos Biology* 2006, **4**(6):1000-1009.

19. Gierl MS, Gruhn WH, von Seggern A, Maltry N, Niehrs C: **GADD45G functions in male sex determination by promoting p38 signaling and Sry expression**. *Dev Cell* 2012, **23**(5):1032-1042.

20. Warr N, Carre GA, Siggers P, Faleato JV, Brixey R, Pope M, Bogani D, Childers M, Wells S, Scudamore CL *et al*: **Gadd45gamma and Map3k4 interactions regulate mouse testis determination via p38 MAPK-mediated control of Sry expression**. *Dev Cell* 2012, **23**(5):1020-1031.

21. Callier P, Calvel P, Matevossian A, Makrythanasis P, Bernard P, Kurosaka H, Vannier A, Thauvin-Robinet C, Borel C, Mazaud-Guittot S *et al*: **Loss of function mutation in the palmitoyl-transferase HHAT leads to syndromic 46,XY disorder of sex development by impeding Hedgehog protein palmitoylation and signaling**. *PLoS Genet* 2014, **10**(5):e1004340.

22. Kuroki S, Matoba S, Akiyoshi M, Matsumura Y, Miyachi H, Mise N, Abe K, Ogura A, Wilhelm D, Koopman P *et al*: **Epigenetic regulation of mouse sex determination by the histone demethylase Jmjd1a**. *Science* 2013, **341**(6150):1106-1109.

23. Meeks JJ, Weiss J, Jameson JL: **Dax1 is required for testis determination**. *Nature Genetics* 2003, **34**(1):32-33.

24. Fujimoto Y, Tanaka SS, Yamaguchi YL, Kobayashi H, Kuroki S, Tachibana M, Shinomura M, Kanai Y, Morohashi K, Kawakami K *et al*: **Homeoproteins Six1 and Six4 regulate male sex determination and mouse gonadal development**. *Dev Cell* 2013, **26**(4):416-430.

25. Weiss J, Meeks JJ, Hurley L, Raverot G, Frassetto A, Jameson JL: **Sox3 is required for gonadal function, but not sex determination, in males and females**. *Molecular and Cellular Biology* 2003, **23**(22):8084-8091.

26. Sutton E, Hughes J, White S, Sekido R, Tan J, Arboleda V, Rogers N, Knower K, Rowley L, Eyre H *et al*: **Identification of SOX3 as an XX male sex reversal gene in mice and humans**. *J Clin Invest* 2011, **121**(1):328-341.

27. Chaboissier MC, Kobayashi A, Vidal VI, Lutzkendorf S, van de Kant HJ, Wegner M, de Rooij DG, Behringer RR, Schedl A: **Functional analysis of Sox8 and Sox9 during sex determination in the mouse**. *Development* 2004, **131**(9):1891-1901.

28. Polanco JC, Wilhelm D, Davidson TL, Knight D, Koopman P: **Sox10 gain-of-function causes XX sex reversal in mice: implications for human 22q-linked disorders of sex development**. *Hum Mol Genet* 2010, **19**(3):506-516.

29. Gautier A, Le Gac F, Lareyre JJ: **The gsdf gene locus harbors evolutionary conserved and clustered genes preferentially expressed in fish previtellogenic oocytes**. *Gene* 2011, **472**(1-2):7-17.

30. Gnessi L, Emidi A, Jannini EA, Carosa E, Maroder M, Arizzi M, Ulisse S, Spera G: **Testicular development involves the spatiotemporal control of PDGFs and PDGF receptors gene expression and action**. *J Cell Biol* 1995, **131**(4):1105-1121.

31. Lee MM, Seah CC, Masiakos PT, Sottas CM, Preffer FI, Donahoe PK, Maclaughlin DT, Hardy MP: **Mullerian-inhibiting substance type II receptor expression and function in purified rat Leydig cells**. *Endocrinology* 1999, **140**(6):2819-2827.

32. Loy CJ, Yong EL: **Sex, infertility and the molecular biology of the androgen receptor**. *Curr Opin Obstet Gynecol* 2001, **13**(3):315-321.

33. Schiffer L, Anderko S, Hannemann F, Eiden-Plach A, Bernhardt R: **The CYP11B subfamily**. *J Steroid Biochem Mol Biol* 2014.

34. Russell DW, Wilson JD: **Steroid 5 alpha-reductase: two genes/two enzymes**. *Annu Rev Biochem* 1994, **63**:25-61.

35. Vainio S, Heikkila M, Kispert A, Chin N, McMahon AP: **Female development in mammals is regulated by Wnt-4 signalling**. *Nature* 1999, **397**(6718):405-409.

36. Boulanger L, Pannetier M, Gall L, Allais-Bonnet A, Elzaiat M, Le Bourhis D, Daniel N, Richard C, Cotinot C, Ghyselinck NB *et al*: **FOXL2 Is a Female Sex-Determining Gene in the Goat**. *Current Biology* 2014, **24**(4):404-408.

37. Chassot AA, Gregoire EP, Magliano M, Lavery R, Chaboissier MC: **Genetics of ovarian differentiation: Rspo1, a major player**. *Sex Dev* 2008, **2**(4-5):219-227.

38. Maatouk DM, DiNapoli L, Alvers A, Parker KL, Taketo MM, Capel B: **Stabilization of beta-catenin in XY gonads causes male-to-female sex-reversal**. *Hum Mol Genet* 2008, **17**(19):2949-2955.

39. Yao HH, Matzuk MM, Jorgez CJ, Menke DB, Page DC, Swain A, Capel B: **Follistatin operates downstream of Wnt4 in mammalian ovary organogenesis**. *Dev Dyn* 2004, **230**(2):210-215.

40. Kashimada K, Pelosi E, Chen H, Schlessinger D, Wilhelm D, Koopman P: **FOXL2 and BMP2 act cooperatively to regulate follistatin gene expression during ovarian development**. *Endocrinology* 2011, **152**(1):272-280.

41. Guiguen Y, Fostier A, Piferrer F, Chang CF: **Ovarian aromatase and estrogens: a pivotal role for gonadal sex differentiation and sex change in fish**. *Gen Comp Endocrinol* 2010, **165**(3):352-366.

42. Dechering K, Boersma C, Mosselman S: **Estrogen receptors alpha and beta: two receptors of a kind?** *Curr Med Chem* 2000, **7**(5):561-576.

43. Hargitai B, Kutnyanszky V, Blauwkamp TA, Stetak A, Csankovszki G, Takacs-Vellai K, Vellai T: **xol-1, the master sex-switch gene in C. elegans, is a transcriptional target of the terminal sex-determining factor TRA-1**. *Development* 2009, **136**(23):3881-3887.

44. Villeneuve AM, Meyer BJ: **The role of sdc-1 in the sex determination and dosage compensation decisions in Caenorhabditis elegans**. *Genetics* 1990, **124**(1):91-114.

45. Trent C, Purnell B, Gavinski S, Hageman J, Chamblin C, Wood WB: **Sex-Specific Transcriptional Regulation of the C-Elegans Sex-Determining Gene Her-1**. *Mech Develop* 1991, **34**(1):43-55.

46. Schedl T, Graham PL, Barton MK, Kimble J: **Analysis of the role of tra-1 in germline sex determination in the nematode Caenorhabditis elegans**. *Genetics* 1989, **123**(4):755-769.

47. Doniach T, Hodgkin J: **A sex-determining gene, fem-1, required for both male and hermaphrodite development in Caenorhabditis elegans**. *Dev Biol* 1984, **106**(1):223-235.

48. Billeter JC, Goodwin SF: **Characterization of Drosophila fruitless-gal4 transgenes reveals expression in male-specific fruitless neurons and innervation of male reproductive structures**. *J Comp Neurol* 2004, **475**(2):270-287.

49. Cline TW: **Evidence that sisterless-a and sisterless-b are two of several discrete "numerator elements" of the X/A sex determination signal in Drosophila that switch Sxl between two alternative stable expression states**. *Genetics* 1988, **119**(4):829-862.

50. Kramer SG, Jinks TM, Schedl P, Gergen JP: **Direct activation of Sex-lethal transcription by the Drosophila runt protein**. *Development* 1999, **126**(1):191-200.

51. Cline TW: **Two closely linked mutations in Drosophila melanogaster that are lethal to opposite sexes and interact with daughterless**. *Genetics* 1978, **90**(4):683-698.

52. Du C, McGuffin ME, Dauwalder B, Rabinow L, Mattox W: **Protein phosphorylation plays an essential role in the regulation of alternative splicing and sex determination in Drosophila**. *Mol Cell* 1998, **2**(6):741-750.
